# *Nebesna sotnia* gen. & sp. nov. from Baltic amber supports a Pangean distribution of the amphinotic family Ameletopsidae (Ephemeroptera)

**DOI:** 10.1038/s41598-025-01722-8

**Published:** 2025-05-26

**Authors:** Roman J. Godunko, Alexander V. Martynov, Jonas Damzen, Arnold H. Staniczek

**Affiliations:** 1 Biology Centre of the Czech Academy of Sciences, Institute of Entomology, Branišovská 31, 37005 České Budějovice, Czech Republic; 2 Faculty of Biology and Environmental Protection, Department of Invertebrate Zoology and Hydrobiology, University of Lodz, Banacha 12/16, 90237 Łódź, Poland; 3 State Museum of Natural History, National Academy of Sciences of Ukraine, Teatralna 18, Lviv, 79008 Ukraine; 4 National Museum of Natural History, National Academy of Sciences of Ukraine, Bohdan Khmelnytsky 15, Kyiv, 01030 Ukraine; 5 Independent Author, Talino Str. 33-1, 05200 Vilnius, Lithuania; 6 Department of Entomology, State Museum of Natural History Stuttgart, Rosenstein 1, 70191 Stuttgart, Stuttgart, Germany

**Keywords:** Mayflies, Siphlonuroidea, Pangea, Laurasia, Mesozoic, Cenozoic, amphinotic distribution, Taxonomy, Zoology, Entomology, Evolution, Palaeontology, Ecology, Biogeography

## Abstract

Extant representatives of the mayfly family Ameletopsidae Edmunds, 1957 as well as other three small families (Nesameletidae Riek, 1973, Oniscigastridae Lameere, 1917 and Rallidentidae Penniket, 1966) traditionally have been classified within the paraphyletic superfamily Siphlonuroidea. Except for Rallidentidae, which are endemic to New Zealand, they have an amphinotic distribution. Ameletopsidae are present with two genera in South America, one genus in Australia, and one genus in New Zealand. The fossil record of Ameletopsidae is scarce. The Mesozoic monospecific genus *Promirara* Jell & Duncan, 1986 was described from a larva of the Early Cretaceous Koonwarra Fossil Bed in Australia. Also associated with Ameletopsidae is the Cenozoic genus *Balticophlebia* Demoulin, 1968, which was recorded from Eocene Baltic amber based on a female adult. While the systematic position of the Eocene *Balticophlebia* seems to be still unclear and in need of clarification, we are able to confirm the presence of Ameletopsidae in the Eocene of Europe by describing *Nebesna sotnia* **gen. & sp. nov.** based on a relatively well-preserved male imago from Baltic amber. The fossil record thus indeed supports an ancient Pangean history of this family.

## Introduction

Within the diverse and widely distributed superfamily Siphlonuroidea, characterized by the maxillae with three dentisetae in larvae, distinctly transversal thoracic mesonotal suture in combination with 5-segmented tarsi of all legs and by CuA and CuP connected basally, containing a series of intercalary veins going towards the hind margin of the forewing in adults, Kluge et al.^1^ distinguished two groups of families allocated to the Northern Hemisphere and the Southern Hemisphere, respectively. While in the Northern Hemisphere families the mesofurcasternal protuberances are contiguous over their entire length, the group of the Southern Hemisphere families is characterized by the presence of mesofurcasternal protuberances, which are separated at least posteriorly by a median invagination due to the anterior shift of the metathoracic ganglion^1,2^. A single exemption is the family Siphluriscidae Zhou and Peters, 2003, with the plesiomorphic genus and species *Siphluriscus chinensis* Ulmer, 1920. The family Siphluriscidae, although showing all the typical characters of Siphlonuroidea, was not included in this taxon by Kluge et al.^1^, because adult characters were not known in detail at that time and only described later by Zhou and Peters^3^. Being distributed in the Northern Hemisphere, namely in Southern China and Vietnam^4^, *S. chinensis* possesses weakly separated mesofurcasternal protuberances at least posteriorly, similarly to the group of the siphlonuroid families of the Southern Hemisphere^1^.

The group of Siphlonuroidea from the Southern Hemisphere includes only four small families, mostly with amphinotic distribution. The family Ameletopsidae Edmunds, 1957 is well known for their carnivorous larvae, which feed on smaller invertebrates such as other mayflies, stoneflies and chironomids^5^. Ameletopsidae include six extant species in three extant genera, namely *Chaquihua* Demoulin, 1955 and *Chiloporter* Lestage, 1931 from Chile and Argentina^6–8^, the Australian genus *Mirawara* Harker, 1954 with three known species in Northern and South East Queensland, New South Wales, and Victoria^9–13^, and the genus *Ameletopsis* Phillips, 1930 with the single species *A. perscitus* (Eaton, 1899), which is widely distributed throughout New Zealand including both Great and Little Barrier Islands^14,15^.

The family Nesameletidae Riek, 1973 likewise shows an amphinotic pattern of distribution^1,16^. Initially established as a monospecific genus by Eaton^17^, the genus *Nesameletus* Tillyard, 1933 was later complemented by five more species, all endemic to New Zealand^15,18–20^. The Australian genus *Ameletoides* Tillyard, 1933 with one species *A. lacusalbinae* Tillyard, 1933 is known from New South Wales, Victoria, and Tasmania. Finally, the genus *Metamonius* Eaton, 1885 with three described species is recorded from South America, mainly from Southern Argentina and Chile^21–25^.

The third siphlonuroid family showing amphinotic distribution is Oniscigastridae Lameere, 1917, with two species in the genus *Oniscigaster* McLachlan, 1873 in New Zealand, the Neotropical monospecific genus *Siphlonella* Needham & Murphy, 1924, and another genus *Tasmanophlebia* Tillyard, 1921 distributed in Australia with three species^14,21,24^. Nevertheless, Kluge^16^ used principles of non-ranking nomenclature, referred *Siphlonella* as taxon subordinated to *Tasmanophlebia* due to common characters in the larval gills, and the MP furcation in the hind wings.

Except for the suggestion by Kluge^16^ to attribute *Siphlonella ventilans* Needham & Murphy, 1924 to the genus *Tasmanophlebia*, there are no amphinotic genera, extending their distribution to more than one region (e.g. South America and Australia/New Zealand), which may indicate an ancient splitting of Gondwanaland, as considered by Barber-James et al.^26^. The same applies to Coloburiscidae Landa, 1973, another non-siphlonuroid family with amphinotic distribution.

Finally, the endemic New Zealand family Rallidentidae Penniket, 1966 includes only a single genus *Rallidens* with two described species^15^. While *Rallidens mcfarlanei* Penniket, 1966 is distributed in the North Island and the northwestern part of the South Island, *Rallidens platydontis* Staniczek & Hitchings, 2014 is restricted to the eastern and southern South Island^27^.

While we have a good overview on the extant diversity of the Southern Hemisphere Siphlonuroidea, markedly less is known about their fossil history. While there is a relatively diverse paleofauna of Siphlonuroidea documented in Eurasian deposits^2^, there are only few fossil records of Siphlonuroidea south of the equator. Apart from the recently established Astraeopteridae Storari et al., 2023 from the Lower Cretaceous Crato Formation in Brazil, there are only three genera of Siphlonuroidea recorded south of the equator. These are bottom-dwelling larvae of three Mesozoic monospecific genera described from the Early Cretaceous Koonwarra Fossil Bed in Australia, which have been initially attributed to Siphlonuridae^28–30^.

*Australurus plexus* Jell & Duncan, 1986 and *Dulcimanna sculptor* Jell & Duncan, 1986, judging on the original description and available images, can be placed within suborder Pisciforma [Tridentiseta sensu Kluge^16^]. However, Kluge^16^ assigned these taxa to Euplectoptera *incertae sedis*. The larvae of *A. plexus* are considered a mass species, which is common in the autochthonous fauna of the Koonwarra Beds, just as the larvae of *Protoligoneuria limai* Demoulin, 1955 (family Hexagenitidae Lameere, 1917) is considered a mass species in the Crato Formation^31,32^. Based on the structure of the gills, Jell & Duncan^28^ believed that *Australurus* is related to the Onscigastrid genus *Tasmanophlebia*. In addition, the larvae of *Australurus* are characterised by a well-developed large head, with markedly enlarged labrum and clypeus, somewhat resembling those in the carnivorous larvae of Ameletopsidae^7,11,16^. However, the cerci of *Australurus* larvae lack dense rows of long swimming setae typical for most Siphlonuroidea. On the other hand, the genus *Dulcimanna* is characterised by well-developed setae on the cerci in combination with relatively large gills^28^. However, the elongated, tube-shaped body of the larva is closer to Baetoidea than to Siphlonuroidea. Grounded on palaeoecological evidence, the Koonwarra paleolake is hypothesized as a shallow, cold, and freshwater water setting^33^. Larvae of Siphlonuroidea are typical inhabitants of cool, mountainous streams, rivers and lakes, so the habitat of the Koonwarra lagerstätte might have been suitable for the development of their diverse fauna, including putative representatives like *Australurus* and *Dulcimanna*.

The third genus established by Jell & Duncan^28^ is *Promirara* with their amazing larvae, which are very likely to be predators. While the systematic position of *Australurus* and *Dulcimanna* remains unclear, there is consensus that *Promirara* belongs to Ameletopsidae, judging by the greatly enlarged and widened head and the mouthparts resembling those of the carnivorous larvae of this family^16,28,30,34^. At the same time, *Promirara* differs from extant Ameletopsidae by the presence of a strongly curved subcostal rib located at some distance from the costal margin in gills I–VII, in addition to the costal rib on all gills^16^.

Finally, Jell & Duncan^28^ reported also two enigmatic larvae from Koonwarra site, provisionally marking them as "Siphlonuridae? gen. nov.", which recently was revised and described as new genus *Koonwarrabaetisca* Godunko & Sroka, 2024 in the family Baetiscidae Edmunds & Traver, 1954^33^.

There are two more records of fossil Ameletopsidae discovered from fossil resins in the Northern Hemisphere. Georges Demoulin^35^ established the monotypic genus *Balticophlebia hennigi* Demoulin, 1968 for a female imago embedded in the Eocene Baltic amber, and Godunko & Sroka^33^ briefly mentioned a personal observation of AHS on the presence of an undescribed female imago in Miocene Dominican amber. In both of these cases, a detailed investigation of the material is necessary. Nevertheless, Demoulin^35^ and later Landa & Soldán^36^ attributed *B. hennigi* to Ameletopsidae based on the venation of fore- and hind wings, the proportion of leg segments, and the presence of a shortened, but not vestigial paracercus.

Thus, among all listed siphlonuroid families of the Southern Hemisphere, only Ameletopsidae have fossil records. Moreover, extinct specimens matching the diagnosis of Ameletopsidae were found both in Mesozoic sediments of the Southern Hemisphere and in Cenozoic resins of the Northern Hemisphere. We here describe the male imago of *Nebesna sotnia* **gen. & sp. nov.** from Baltic amber and assign it to Ameletopsidae, as it matches the diagnosis of its winged stages. This finding thus may support a wider distribution of this family along with an older age than previously assumed, apparently dating back to the time before the breakup of Pangaea (215–175 My).

## Results

### Systematic palaeontology

Class Insecta Linnaeus, 1758

Subclass Pterygota Lang, 1888

Order Ephemeroptera Hyatt & Arms, 1891

Family Ameletopsidae Edmunds, 1957

*Nebesna* **gen. nov.**

LSID urn:lsid:zoobank.org:act:E09D9228-1C1B-4EC2-9C01-F747675B6BF0

#### Derivation of name

The generic name *Nebesna* **gen. nov.** is of female gender and refers to the tragic events in Ukraine during the Revolution of Dignity in 2013–2014, honouring the victims of Nebesna Sotnia [*Heбecнa Coтня*; *the Heavenly Hundred*] and all victims of the Russian military aggression.

#### Type species

*Nebesna sotnia* **sp. nov.**

#### Species composition

Monospecific.

#### Diagnosis

***Male imago:*** *Body and forewing* (**i**) small, 7.28 mm and 7.80 mm in length respectively [as preserved]; *Head* (**ii**) lower portion of compound eyes distinctly narrow; *Thorax* (**iii**) mesonotal suture short, directed backward medially; furcasternal protuberances strongly widened posteriorly; *Wings* (i**v**) *forewing* relatively narrow, with basitornal margin short, as long as 0.56 of tornoapical margin length; tornus placed approximately 0.36 from the wing base; iRS arise from RSa_1_; MP forked after 0.25 of its length; 4 simple stout intercalary veins in cubital field not accompanied with basally free intercalaries; distal ends of CuP and A_1_ approximated; (**v**) *hind wing* as long as 0.40 of forewing, with costal process acutely pointed apically; tornoapical margin without fold; MA–RSp fork situated after 0.13 of vein base; cross veins moderately developed, lacking in anal field; basally connected veins poorly developed.

*Nebesna sotnia* **sp. nov.**

LSID urn:lsid:zoobank.org:pub:14D53A7F-C483-4A12-B973-EAFD6A5B282E

**Fig. 1:**
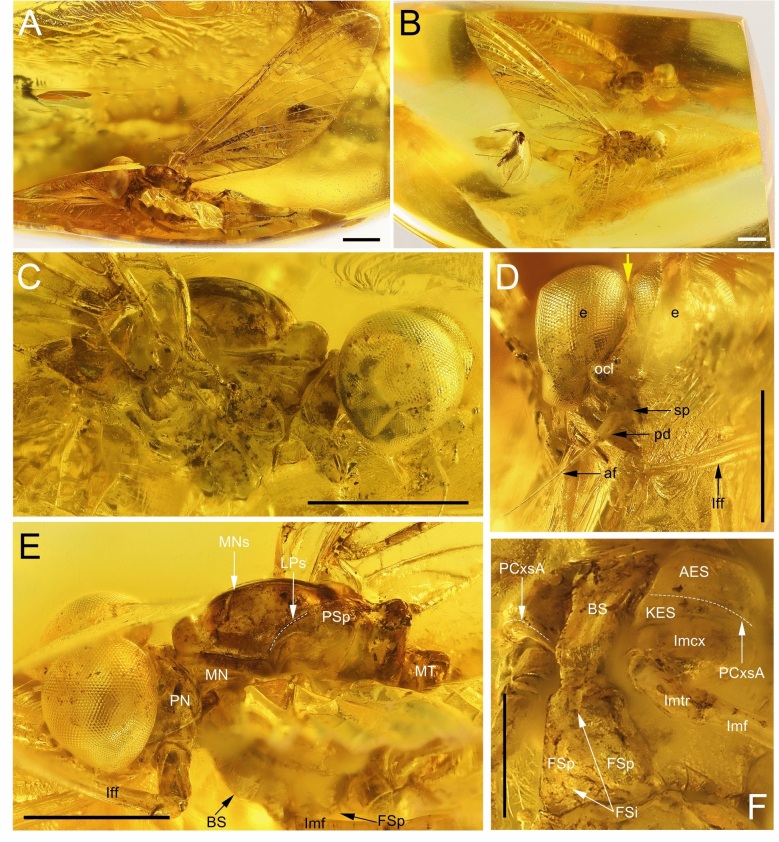
*Nebesna sotnia* **gen. et sp. nov.** (**A**–**F**), holotype, male imago; Eocene Baltic amber [NMNH NASU, catalogue number IKOFZ-Ib 8921, Kyiv, Ukraine]. (**A**) enlarged total lateral view from left side; (**B**) entire piece of amber with embedded holotype in lateral view from right side; (**C**) head and thorax in lateral view from right side; (**D**) head in frontal view; (**E**) head and thorax in lateral view from left side; (**F**) thorax in ventrolateral view from right side. Scale bars 1 mm (**A**–**E**), 0.5 mm (**F**). Abbreviations. *Head*: af—antennal flagellum; e—eyes; ocl—lateral ocellus; pd—pedicle; sp—scape. *Thorax*: lff—left forefemur; lmcx—left middle coxa; lmf—left middle femur; lmtr—left middle trochanter; PN—pronotum; MT—metanotum; *Mesothorax*: AES—anepisternum; BS—basisternum; FSi—furcasternal impression; FSp—furcasternal protuberance; KES—katepisternum; LPs—lateroparapsidal suture; MN—mesonotum; MNs—mesonotal suture; PCxsA—anterior paracoxal suture; PSp—posterior scutal protuberance. Nomenclature of thoracic structures used throughout the text is based on Kluge et al.^1^ and Kluge^16^. Contiguous medially compound eyes are indicated by yellow arrow. White dashed line is indicated of the lateroparapsidal suture [LPs] on the (**E**) and the anterior paracoxal suture [PCxsA] on the (**F**).

**Fig. 2:**
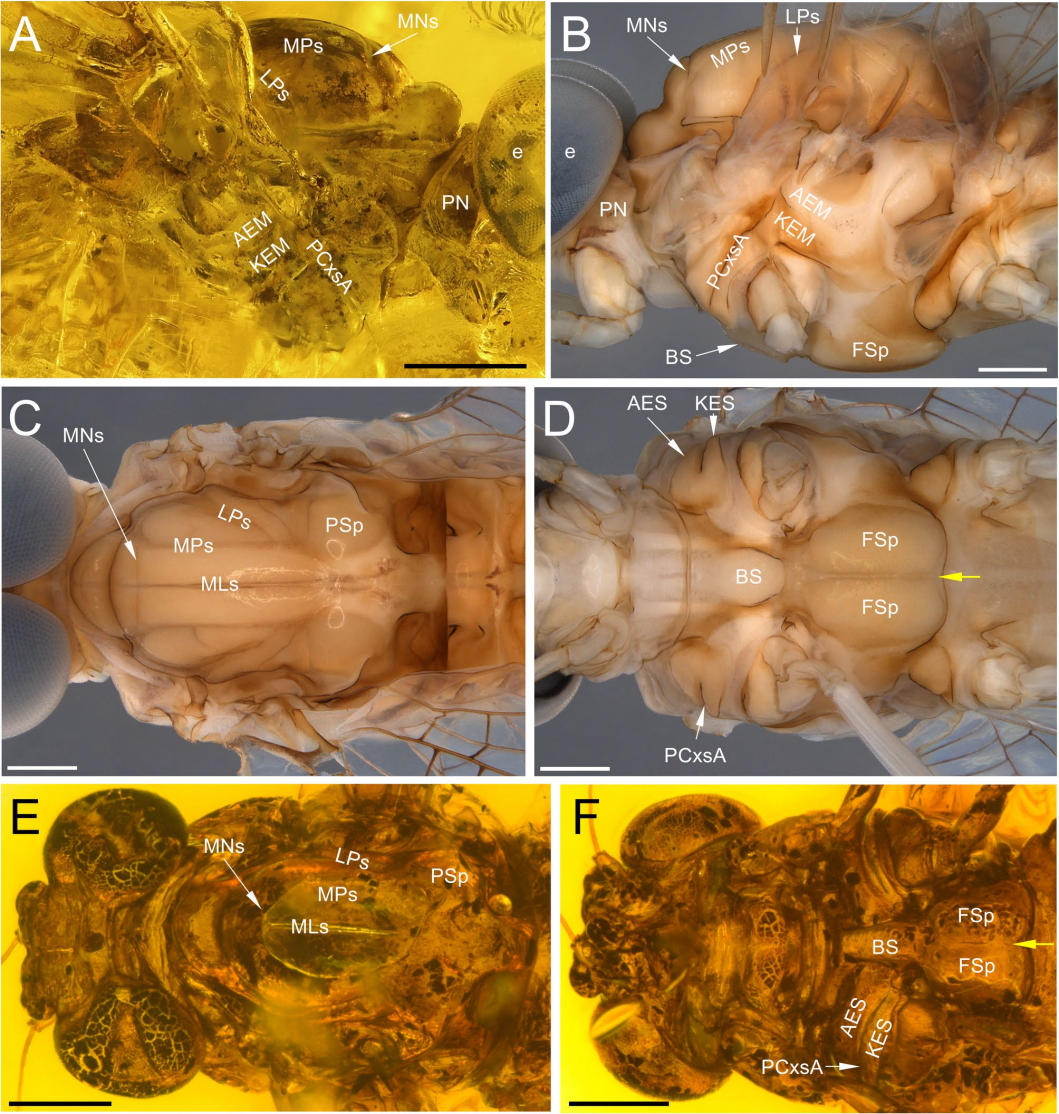
*Nebesna sotnia* **gen. et sp. nov.** (**A**) [holotype, male imago], comparative extant material of *Rallidens mcfarlanei* Penniket, 1966 (**B**–**D**) [male imago; family Rallidentidae; New Zealand; SMNS coll.] and comparative extinct material of *Siphloplecton* sp. (**E**, **F**) [female imago; Metretopodidae; Eocene Baltic amber; Stantien & Becker coll. in Geowissenschaftliches Zentrum coll., Universität Göttingen, Germany]. Scale bars 0.5 mm. Abbreviations. Head: e—eyes. Thorax: PN—pronotum; Mesothorax: AEM—anepimeron; AES—anepisternum; BS—basisternum; FSi—furcasternal impression; FSp—furcasternal protuberance; KEM—katepimeron; KES—katepisternum; LPs—lateroparapsidal suture; MLs—median longitudinal suture; MN—mesonotum; MNs—mesonotal suture; MPs—medioparapsidal suture; PCxsA—anterior paracoxal suture; PSp—posterior scutal protuberance. The border between furcasternal protuberances is indicated by yellow arrow.

**Fig. 3:**
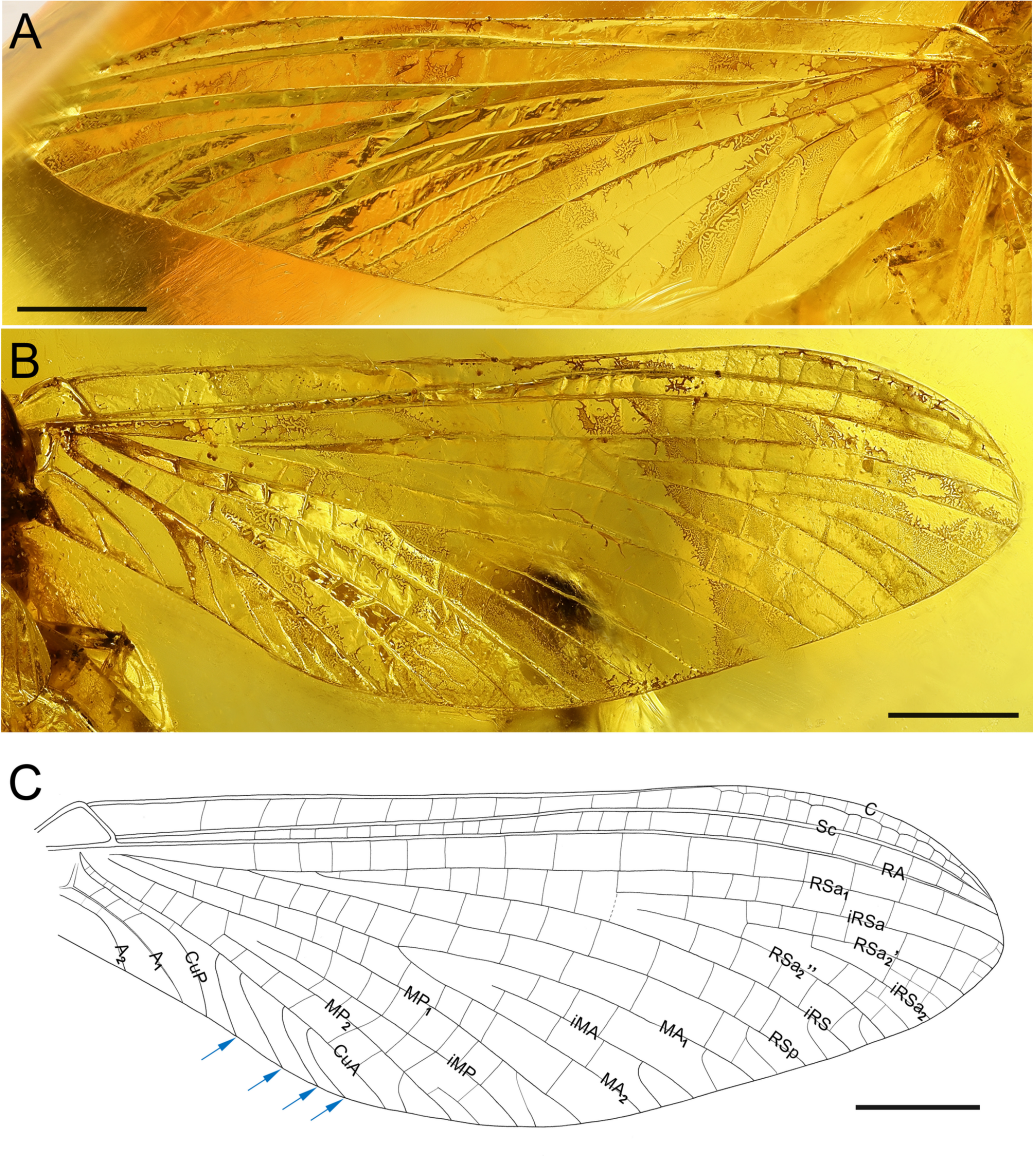
*Nebesna sotnia* **gen. et sp. nov.** (**A**–**C**) [holotype, male imago]; right forewing ventrally (**A**) and dorsally (**B**, **C**). Scale bars 1 mm. Stout simple intercalaries [iCu] are indicated by blue arrows. Nomenclature of the forewing veins used throughout the text is based on Kluge^16^.

**Fig. 4:**
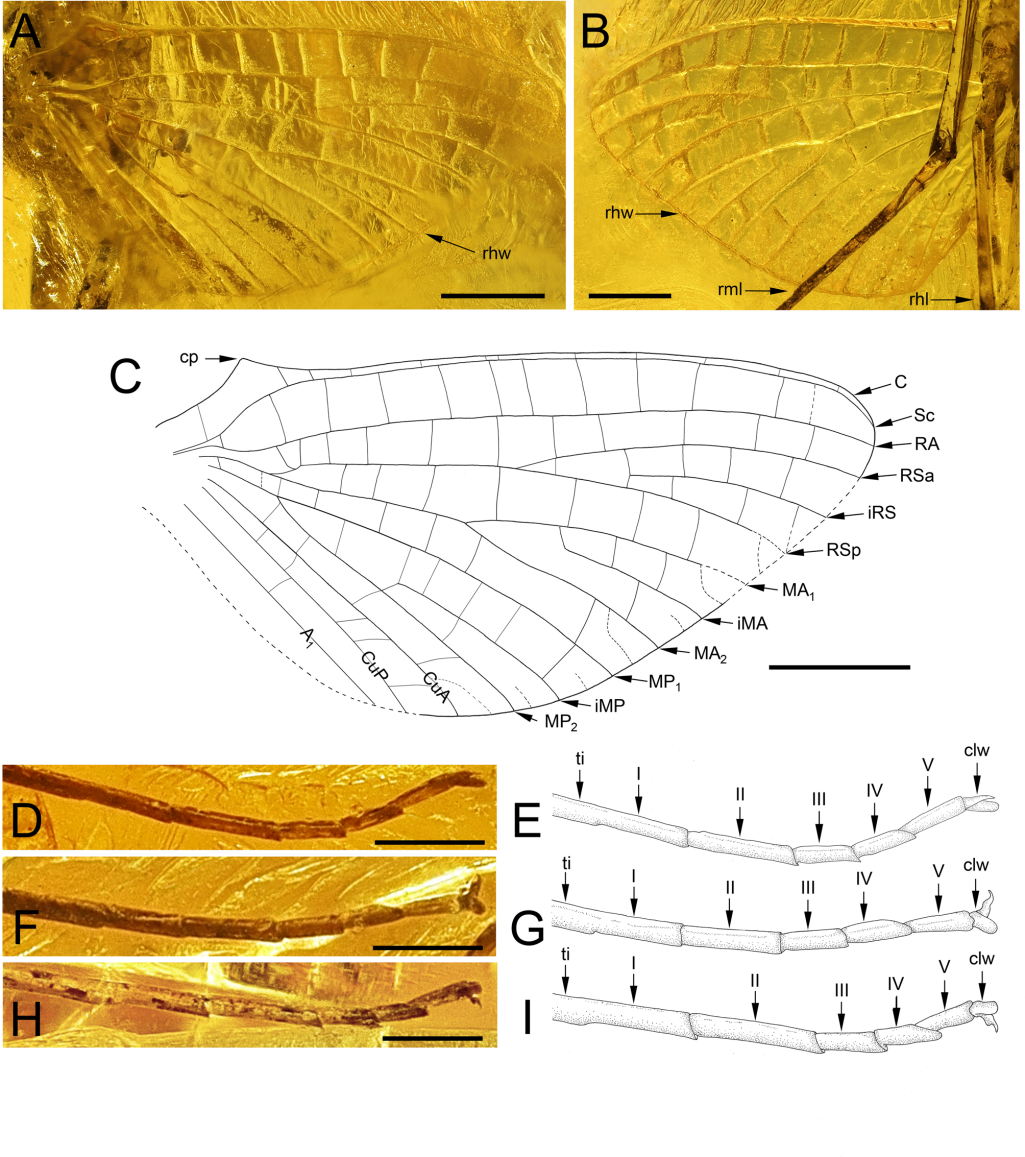
*Nebesna sotnia* **gen. et sp. nov.** (**A**–**I**) [holotype, male imago]; (**A**, **C**) right hind wing dorsally; (**B**) right hind wing ventrally; (**D**, **E**) right middle leg; (**F**, **G**) right hind leg; (**H**, **I**) hind left leg. Scale bars 0.5 mm (**A**–**C**), 0.25 mm (**D**, **F**, **H**), without scales (**E**, **G**, **I**). Abbreviations. *Right hind wing*: cp—costal process; *Legs*: I–V—tarsal segments; clw—tarsal claws; rhw—right hind wing; rml—right middle leg; rhl—right hind leg; ti—tibia. Nomenclature of the hind wing veins used throughout the text is based on Kluge^16^.

Figures 1, 2, 3 and 4, Table 1, Supplementary Table S1

**Table 1:** Measurements of the holotype of *Nebesna sotnia* **gen. & sp. nov.** (male imago; NMNH NASU, catalogue number IKOFZ-Ib 8921, Kyiv, Ukraine).

Characters	mm	Characters	mm
Length of body	7.28*	Length of tibia	0.73*
Length of right foreleg	3.13*	Length of tarsus	–
Length of femur	1.71	Segment I	–
Length of tibia	1.31	Segment II	–
Length of tarsus	0.11*	Segment III	–
Segment I	0.11*	Segment IV	–
Segment II	–	Segment V	–
Segment III	–	Length of right hind leg	3.48
Segment IV	–	Length of femur	1.30
Segment V	–	Length of tibia	1.15
Length of left foreleg	1.61*	Length of tarsus	1.04
Length of femur	1.61*	Segment I	0.20
Length of tibia	–	Segment II	0.30
Length of tarsus	–	Segment III	0.19
Segment I	–	Segment IV	0.14
Segment II	–	Segment V	0.20
Segment III	–	Length of left hind leg	3.58
Segment IV	–	Length of femur	1.32
Segment V	–	Length of tibia	1.16
Length of right middle leg		Length of tarsus	1.10
Length of femur	1.18	Segment I	0.23
Length of tibia	1.08	Segment II	0.33
Length of tarsus	1.02	Segment III	0.19
Segment I	0.23	Segment IV	0.15
Segment II	0.26	Segment V	0.20
Segment III	0.18	Length of right forewing	7.80
Segment IV	0.16	Length of left forewing	0.68*
Segment V	0.19	Length of right hind wing	2.63
Length of left middle leg	1.92*	Length of left hind wing	1.53*
Length of femur	1.19	Hind/Fore wings length ratio	0.34

*—preserved part.

#### Type material

**Holotype:** Male imago in Baltic amber, Middle Eocene (35–47 million years); formerly in Jonas Damzen collection (Vilnius, Lithuania; https://www.amberinclusions.eu; previous inventory number JDC13363); now housed in the collection of the National Museum of Natural History, National Academy of Sciences of Ukraine (Kyiv, Ukraine), under inventory number IKOFZ-Ib 8921.

#### Derivation of name

The species name “sotnia” [*coтня; hundred*] is part of the term “Nebesna Sotnia”, which refers to the victims of Ukrainian Revolution of Dignity in 2013–2014, with deep gratitude for the feat, bravery and selflessness of all Ukrainians who suffered from Russian military aggression. The species name is a female noun in apposition.

#### Diagnosis

***Male imago:*** As for *Nebesna* **gen. nov.**, as monospecific.

#### Generalities

Relatively well-preserved imaginal specimen in clear, translucent amber, better visible from the left in lateral aspect, both forelegs incomplete, foretarsi missing; distal part of left middle leg missing; abdominal sternite IX, entire segment X, male genitalia, and cerci missing. Only right fore- and hind wings well-preserved and complete; left wings twisted, partly damaged and covered by multiple resin layers.

The specimen is clearly defined as an imago because of the translucent and hyaline fore- and hind wings, and the absence of microtrichia along their posterior margin.

The entire piece of amber with numerous cracks, organic debris, and resin layers. In the same piece of amber, an adult specimen of Sciaridae (Diptera) is embedded (Fig. 1A, B).

#### Description

***Male imago*** (Figs. 1, 2, 3 and 4; Table 1; Supplementary Table S1). *Colours*. Preserved colour of specimen is yellowish to brown and darker brown. Eyes pale, yellow to dirty yellow; facial keel the same colour; small blackish maculae on ocelli. Thorax darkest, brown to dark brown, paler laterally; irregular artificial blackish maculae scattered on whole surface; the same maculae on base of wings [*result of fossilisation*]; lateral sides of thorax covered by a cloud of turbidity (so-called “Verlumung”). Wings pale, hyaline, translucent, yellow to light brown; venation yellow; cross venation poorly visible; right forewing covered by irregular artificial brownish maculae [*result of fossilisation*]. Legs yellow to dark brown, darkened distally; irregular artificial blackish maculae scattered on surface of preserved segment. Abdominal segments yellow, partly translucent.

*Measurements*. Body length 7.28 mm [*as preserved*]; forewing length 7.80 mm; hind wing length 2.63 mm. Maximum forewing width 0.34 × maximum length; hind wing 0.34 × of forewing length. For other measurements see Table 1.

*Head*. Facial keel relatively small, not protruding anteriorly. Antennae pale, yellow to dirty yellow. Frontal and left lateral ocellus poorly visible due to resin layers, all without conspicuous coloration. Compound eyes divided into two portions; upper portion of compound eyes well developed, large and widely rounded, contiguous medially; lower portion of each eye approximately 0.62 × as long as the upper portion; border between upper and lower portions of compound eyes distinguishable; facets of compound eyes hexagonal (Fig. 1C–E). No projection on vertex (Fig. 1C).

*Thorax*. Thoracic terga darker than pleura, yellowish-brown to dark brown; pleurae light brown. No traces of specific pigmented areas on mesonotum. Thoracic sterna paler than terga, light brown. Mesonotal suture [MNs] short, shallow, V-shaped, stretched backward medially; medioparapsidal suture [MPs] nearly straight, slightly curved inward posteriorly; lateroparapsidal suture [LPs] relatively short and straight, not curved laterally and not reaching posterior scutal protuberance [PSp] (Figs. 1E, 2A). Anterior paracoxal suture of mesothorax [PCxsA] complete, running across ventral side of episternum clearly separating it into anepisternum [AES] and katepisternum [KES], and terminates reaching sternum (Fig. 2A). Basisternum of mesonotum [BS] slightly elongated; furcasternal protuberances [FSp] not contiguous, separated by a median impression; this median furcasternal impression [FSi] is widened posteriorly (Fig. 1F).

*Wings*. Forewings hyaline, translucent, not frosted. Cross veins paler than longitudinal veins. Cross veins and small intercalary veins moderately developed, whitish yellow to yellow, poorly recognizable along tornoapical margin (Fig. 3).

Pterostigmatic area translucent; a cluster of up to 13 forked cross veins in pterostigmatic area. Cubital brace well preserved, strongly arched. C, Sc, and RA well visible throughout their length. RS forked near base, approximately at 0.15 of its length; cross venation moderately developed; iRS arises from RSa_1_ close to RS fork; intercalary venation between RSa_1_ and iRS well-developed; RSa_2_’’ short, free basally; RS sector with a few intercalaries connected basally. MA symmetrically forked at 0.45 of its length; MA_1_ and MA_2_ connected to iMA by 3 cross veins from each side. MP asymmetrical, forked after 0.25 of its length; MP_2_ approximated to CuA, iMP approximated to MP_2_; iMP shorter than MP_2_, connected to MP_1_ and MP_2_ by numerous cross veins; several basally connected intercalaries in MA and MP fields. Cubital field relatively narrow and short; four stout, simple intercalaries [iCu] of different length running from CuA towards posterior margin of wing, terminating at basitornal margin; the length of iCu increases from iCu_1_ to iCu_4_ respectively; no cross veins and small intercalaries between main stout cubital intercalaries; approximately 7 small cross veins between CuA and CuP situated strongly basally; CuA and CuP connected near wing base; CuA and CuP smoothly curved toward wing base; tornus close to CuA, weakly pronounced, located at about 0.36 of forewing length from base; CuP slightly bent distally; at least two relatively straight veins in anal field; A_1_ basally approximating CuP; three visible cross veins between CuP and A_1_; two visible cross veins in anal field between A_1_ and A_2_ (Fig. 3).

*Hind wing* hyaline, translucent, triangular-shaped, relatively wide and not elongated, clearly narrowed anteriorly, as wide as 0.52 of wing length, distinctly narrowed and moderately rounded distally. Longitudinal venation yellow to light brown; cross venation the same colour, moderately developed, hardly recognizable in anal field. Costal process of hind wing well developed, prominent, acute apically; one cross vein in the sector of costal process and humeral angle of hind wing. C and Sc approximated. RSp basally connected to MA; several cross veins between MA_1_ and RSp; MA forked at approximately midlength; MP forked strongly basally, at approximately 0.19 of the distance from vein base. CuA, CuP and A_1_ not forked; venation of anal field poorly recognizable. Small marginal veins recognizable between RS and CuA (Fig. 4A–C).

*Legs*. Forelegs paler than middle and hind legs. Artificial dark brown to blackish maculae covered whole leg surface [*as a result of fossilization*]. Tibiopatellar suture present on basal 1/3 length of middle and hind legs. First tarsomere of middle and hind legs markedly elongated, as long as 0.23 / 0.26 of tibia length respectively; first tarsomere of middle and hind legs fused with tibia. Pretarsal claws of each leg with outer claw hooked and inner claw blunt (Fig. 4D–I).

*Abdominal segments* I–VIII completely preserved; segment IX incomplete; segment X missing; segments almost translucent, yellow; first two segments darkest, light brown; no prominent posterolateral projections on abdominal segments. Genitalia and cerci missing (Fig. 1).

## Discussion

### Systematic placement of *Nebesna* gen. nov.

Based on the significantly reduced size of its hind wings and a costal brace anteriorly fused with the costal vein, *Nebesna* **gen. nov.** is clearly nested within the crown-group Ephemeroptera. *Nebesna* **gen. nov.** has an anteritornous forewing, i.e. the wing tornus is situated between CuA and CuP, as in the vast majority of mayflies ^16^.

**Fig. 5:**
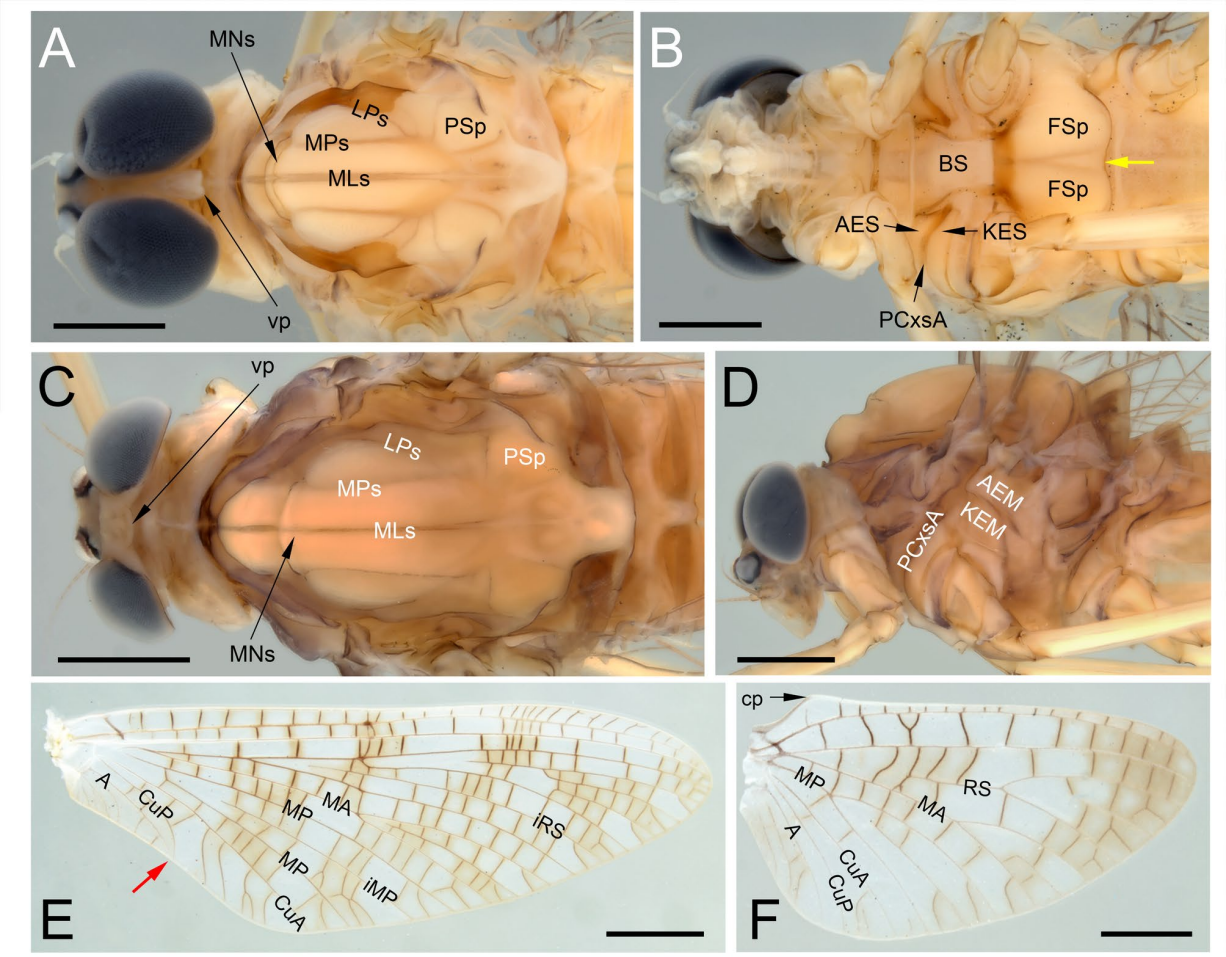
Comparative extant material of *Nesameletus* sp. (**A**–**F**); male imago (**A**, **B**, **E**, **F**), female imago (**C**, **D**) [Nesameletidae; New Zealand; SMNS coll.]. (**A**, **C**) head and thorax in dorsal view; (**B**) head and thorax in ventral view; (**D**) head and thorax in lateral view from left side; (**E**) right forewing in dorsal view; (**F**) right hind wing in dorsal view. Scale bars 1 mm (**A**–**D**), 2 mm (**E**), 1 mm (**F**). Abbreviations. *Head*: vp—vertex projection. *Thorax* (*mesothorax*): AEM—anepimeron; AES—anepisternum; BS—basisternum; FSp—furcasternal protuberance; KEM—katepimeron; KES—katepisternum; LPs—lateroparapsidal suture; MLs—median longitudinal suture; MNs—mesonotal suture; MPs—medioparapsidal suture; PCxsA—anterior paracoxal suture; PSp—posterior scutal protuberance. *Hind wing*: cp—costal process. The border between furcasternal protuberances is indicated by yellow arrow; the distance between distal ends of CuP and A_1_ is indicated by red arrow.

**Fig. 6:**
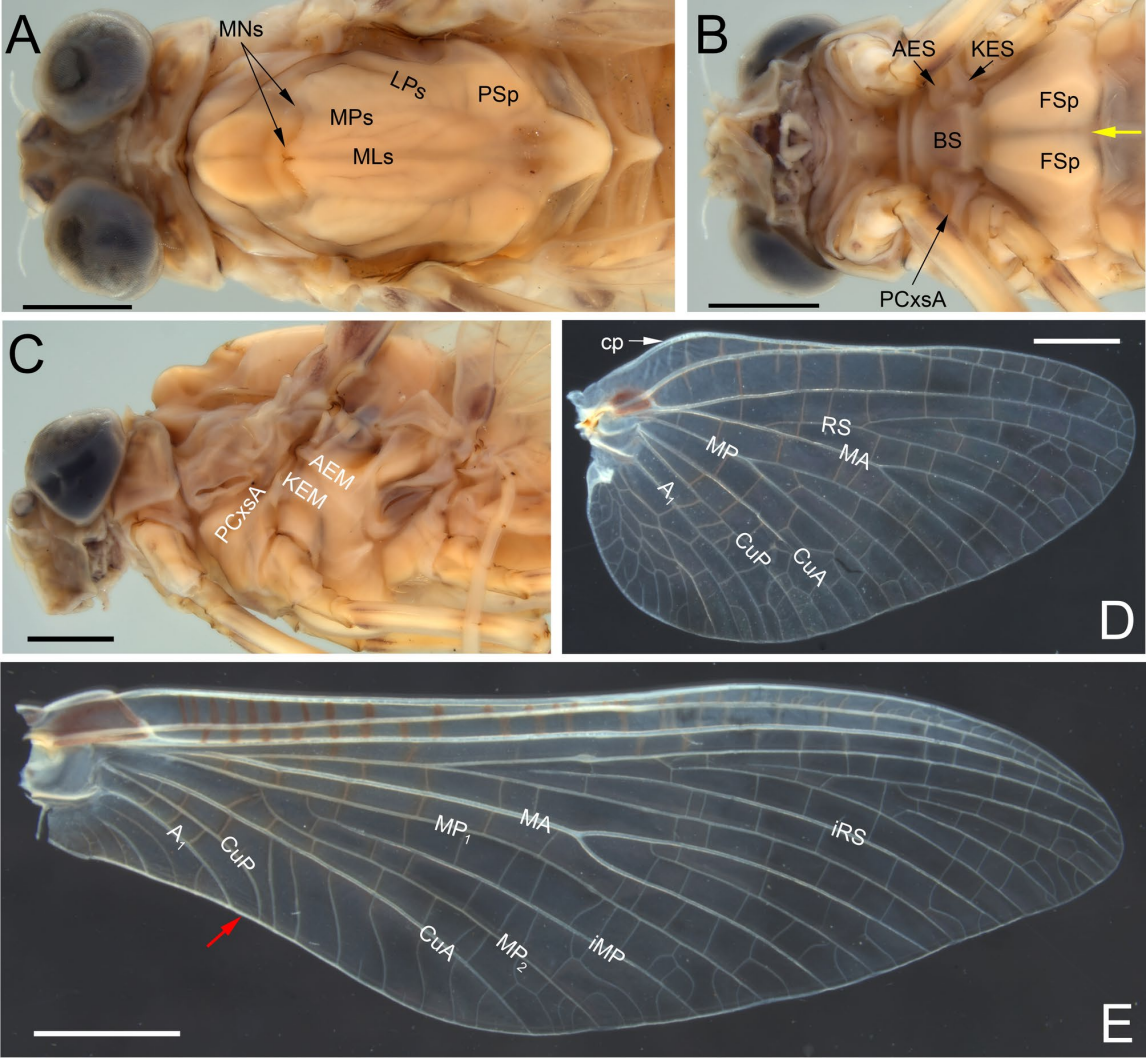
Comparative extant material of *Oniscigaster distans* Eaton 1899; female imago [Oniscigastridae; New Zealand; SMNS coll.]. (**A**) head and thorax in dorsal view; (**B**) head and thorax in ventral view; (**C**) head and thorax in lateral view from left side; (**D**) right hind wing in dorsal view; (**E**) right forewing in dorsal view. Scale bars 1 mm (**A**–**D**), 2 mm (**E**). Abbreviations. *Thorax* (*mesothorax*): AEM—anepimeron; AES—anepisternum; BS—basisternum; FSp—furcasternal protuberance; KEM—katepimeron; KES—katepisternum; LPs—lateroparapsidal suture; MLs—median longitudinal suture; MNs—mesonotal suture; MPs—medioparapsidal suture; PCxsA—anterior paracoxal suture; PSp—posterior scutal protuberance. *Hind wing*: cp—costal process. The border between furcasternal protuberances is indicated by yellow arrow; the distance between distal ends of CuP and A_1_ is indicated by red arrow.

The forewing venation in *Nebesna* **gen. nov.** corresponds to the venation present in Siphlonuroidea (Fig. 3A–C). The fossil taxon shares with Siphlonuroidea a characteristic series of sinusoid, long, intercalary veins in the cubital field, which are occasionally forked towards the hind margin of the wing (see also Fig. 5E, F; 6DE). Additionally, in the male imago of *Nebesna* **gen. nov.**, the first tarsomeres of the middle and hind legs are neither shortened nor fused with tibia, which precludes a placement within the superfamilies Ephemerelloidea, Caenoidea, Leptophlebioidea, and Ephemeroidea, which are all attributed to Furcatergalia sensu Kluge^16^, or to Heptagenioidea (Branchitergalia sensu Kluge^16^, or Setisura sensu McCafferty^37^). Moreover, the attribution of *Nebesna* **gen. nov.** to Heptagenioidea can be excluded due to the well-developed posterior arc of the prealar bridge (see Fig. 1C and fig. 5 (PAB:PA) in Kluge^16^), and due to the presence of a complete anterior paracoxal suture reaching the sternum (PCxsA in Figs. 1F, 2F, 5B, D, 6B, C). This matches the condition in most families of Siphlonuroidea except of Rallidentidae, where the anterior paracoxal suture is incomplete (see below; Fig. 2D), and MNs is nearly transverse (Fig. 2C). We also exclude a placement of in *N. sotnia* **sp. nov.** within Baetoidea due to the five-segmented tarsi in all legs of this fossil species, and also due to the combination of a well-developed mesonotal suture and the basal approximation of CuA to CuP in the forewing (Figs. 1C, E, 2A, 3A–C).

Obviously, the combination of diagnostic characters of *Nebesna* **gen. nov.** excludes this species from most of the presently recognized higher taxa within Ephemeroptera. However, considering wing venation, thoracic sutures, and middle and hind legs, the respective character states are present in Siphlonuroidea.

While the families, which have been attributed to Siphlonuroidea^1^, are each monophyletic due to certain larval autapomorphies, the different lineages share only symplesiomorphic characters between each other^2^. There is also strong molecular evidence that Siphlonuroidea is a paraphyletic assemblage of basal taxa^38,39^. Independently from the paraphyly of Siphlonuroidea, it is also difficult to assign fossil adults like *Nebesna* **gen. nov.** unambiguously to a specific family within Siphlonuroidea, as the apomorphic characters to prove this are mostly present in the larval stages. At best we can say that *Nebesna* **gen. nov.** matches the combination of diagnostic characters of a certain family, which may not necessarily include autapomorphies. Dividing the Siphlonuroidea into two family groups, Kluge et al.^1^ and Kluge^16^ noted in the Northern Hemisphere group the presence of contiguous furcasternal protuberances over their entire length. Other features like the venation of the cubital field and the paracercus are highly variable and considerably overlapping with those of the Southern Hemisphere families (Supplementary Table S1). Nevertheless, the structure of furcasternal protuberances is differing in both these groups, and *Nebesna* **gen. nov.** clearly shows separated furcasternal protuberances like present in the Southern amphinotic families (compare Figs. 1F, 2D, F, 5B, 6B).

There are even further distinguishing characters between *Nebesna* **gen. nov.** and the Northern Hemisphere group of families: The family Siphlonuridae Banks, 1900 differs by the structure of pretarsal claws, which are pointed on all legs. Siphlonuridae also possess remarkable apomorphies in the structure of the male and female copulatory apparatus (for more details see^1,16,40^, which, however, cannot be used for our comparative analysis because the genitalia of *N. sotnia* **sp. nov.** are not preserved. The genus *Dipteromimus* McLachlan, 1875 (Dipteromiminae: Siphlonuridae) was placed by Kluge et al.^1^ in the monogeneric family Dipteromimidae Kluge et al., 1995. It differs well from all other Siphlonuroidea including *Nebesna* **gen. nov.** by the unique combination of larval and adult characters. Within adult apomorphies there are highly reduced hind wings with unforked RS, MA and MP veins, as long as about 0.1 forewings length; forewings not triangular shaped, but markedly narrow proximally, nearly ellipsoidal centrally and distally (pp. 213–214, pl. XX, fig. 35 in^41^; pp. 212–216, fig. 21 in^42^; pp. 1251–1253, figs. 2A, B; 3A, B in^43^). We can also exclude the family Ameletidae McCafferty, 1991, which has an elongated lateroparapsidal suture of mesonotum in combination with the presence of a membranous area between the mesothorcic anepimeron and katepimeron. In a similar way we can also exclude Metretopodidae Traver, 1935 based on the structure of mesonotum and mesosternum (Fig. 2E, F), as well as Acanthametropodidae Edmunds, 1963 and Ametropodidae Bengtsson, 1913 due to significant differences in the venation of wings, namely in the structure of cubital field of forewing and shape of vein triads of hind wings^1,16^.

The Mesozoic family Siphlonephemerellidae Chen & Zheng, 2023 assigned to the superfamily Siphlonuroidea has been established for a single male imago described as *Siphlonephemerella mupengxui* Chen & Zheng, 2023 from Burmese amber^44^. Judging on the structure of the wings venation, tarsomere proportions and structure of the pretarsal claws of the legs, the Cretaceous *Siphlonephemerella* can be clearly distinguished from *Nebesna* **gen. nov.**

Finally, the family Siphluriscidae was formally included to Siphlonuroidea based on larval characters at a time when the adults had not been described^1^. These primitive mayflies were recovered as sister taxon to all other Ephemeroptera s.str. according to the results of combined phylogenetic analysis performed by Ogden et al.^39^. Together with other mayflies, *Nebesna* **gen. nov.** differs from *Siphluriscus* (Siphluriscidae) by the spatial arrangement of the costal brace (see fig. 2a in^45^).

Defining the systematic position of the new fossil genus among the Southern Hemisphere group of families, it should be noted that *Nebesna sotnia* **sp. nov.** lacks the medial projection of the vertex, which is listed as an apomorphy of Nesameletidae, allowing to exclude this family in our analysis (Figs. 1C, D, 5A, C; Supplementary Table S1; see also pp. 115–119, fig. 46 in^1^; pp. 105–108, fig. 30C in^16^). Moreover, the pretarsal claws are similarly pointed on all legs in Nesameletidae, while *Nebesna* **gen. nov.** bears on each leg the outer claw hooked, and the inner claw blunt (Supplementary Table S1).

The anterior paracoxal suture of *Nebesna* **gen. nov.** is complete as in most Siphlonuroidea, which excludes the family Rallidentidae with an apomorphic condition of this character, namely an incomplete PCxsA, which is partly separating the episternum into anepisternum and katepisternum (Fig. 2B, D; pp. 119–120, fig. 51; pp. 120–122, fig. 36C in^16^), and MNs nearly transverse (Fig. 2C; the same in *Siphloplecton* in Fig. 2E). Additionally, Rallidentidae differ from the new fossil genus by the strongly distal position of MP fork in the hind wing (Supplementary Table S1), which is also the case in some Oniscigastridae (the genus *Tasmanophlebia*; see^18,46,47^). Additionally, Oniscigastridae can be easily distinguished by the medially separated compound eyes (see Fig. 6A, in contrast to medially contiguous eyes in *Nebesna* **gen. nov.** showed in Fig. 1D), and the length of first tarsomere of hind leg in males, which is almost as long as the tibia (Supplementary Table S1; the first tarsomere of hind leg as long as 0.25–0.27 of tibia length in the male imago of *N. sotnia* **sp. nov.**).

**Fig. 7:**
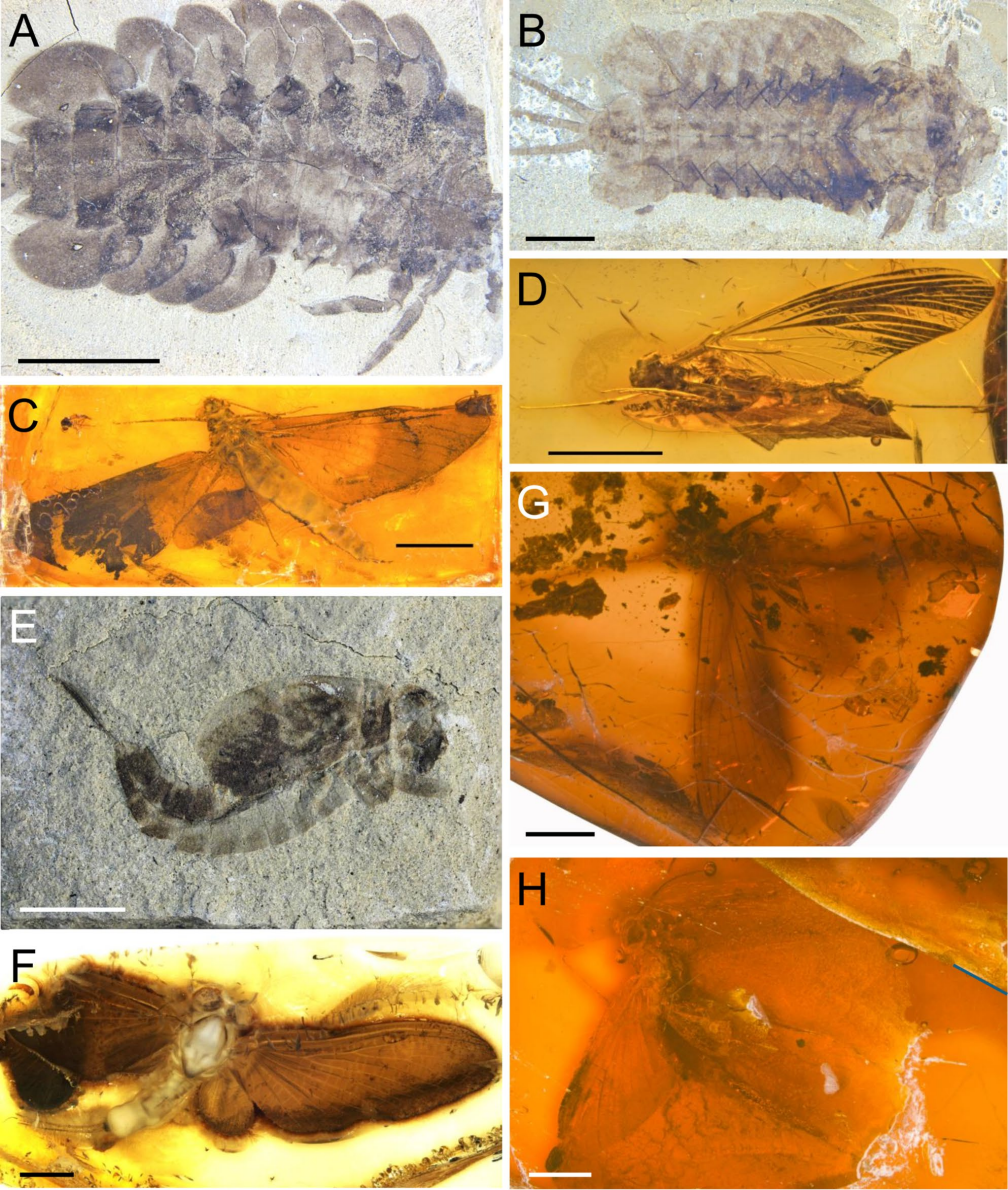
Mayfly taxa with Pangean origin: extinct representatives of the families Ameletopsidae (**A**–**D**), Baetiscidae (**E**, **F**) and Coloburiscidae (**G**, **H**)^33^. (**A**) *Promirara cephalota* Jell & Duncan, 1986, holotype, larva, NMVP102472 A, Early Cretaceous Koonwarra Fossil Bed, Museum Victoria coll., Melbourne (Australia); (**B**) *Promirara cephalota* Jell & Duncan, 1986, paratype, larva, NMVP102473 A, the same site and collection as holotype, scale bar = 2 mm; (**C**) *Balticophlebia hennigi* Demoulin, 1968, holotype, female imago, reg. no. 36, Eocene Baltic amber, Kaliningrad, Russian Federation, Geowissenschaftliches Zentrum coll., Universität Göttingen (Germany); (**D**) Ameletopsidae, gen. et sp. indet., female imago, Do-1268-K, Miocene Dominican amber, Dominican Republic, SMNS coll., Stuttgart (Germany); (**E**) *Koonwarrabaetisca jelli* Godunko & Sroka, 2024, holotype, larva, P103210B, Early Cretaceous Koonwarra Fossil Bed, Museum Victoria coll., Melbourne (Australia); (**F**) *Balticobaetisca bispinata* Staniczek et al. 2022, holotype, male subimago, BaB 1373/1, Eocene Baltic amber, unknown deposit, Christel and Hans Werner Hoffeins coll., Hamburg (Germany); (**G**) *Cronicus major* Demoulin, 1968, holotype, female imago, MB.I.2249, Eocene Baltic amber, Kaliningrad, Russian Federation, Museum für Naturkunde, Berlin (Germany); (**H**) *Cronicus anomalus* (Pictet, 1856), paralectopyte, male subimago, MB.I.2254, Eocene Baltic amber, Kaliningrad, Russian Federation, Museum für Naturkunde, Berlin (Germany). Scale bars 5 mm (**A**, **C**), 2 mm (**B**, **D**, **E**, **F**, **H**), 2.5 mm (**G**).

The adults of the fossil family Astraeopteridae from the Lower Cretaceous Crato Formation are well differing from *Nebesna* **gen. nov.** by their most robust pterothorax, which is wider than the prothorax, and the entire thorax is markedly elevated above the head in a straight line posteriad^2^. Additionally, the Mesozoic genus *Promirara* from Australian Koonwarra Fossil Bed definitely belonging to Ameletopsidae is excluded from the analysis as it is known only from larvae (Figs. 7A, B; see also^28^).

We attribute *Nebesna* **gen. nov.** to Ameletopsidae (see below; Supplement Table S1), based on the unique combination of following imaginal characters: *Head* (**i**) vertex without projection; compound eyes contiguous medially; *Thorax* (**ii**) mesonotal suture shortly stretched backward medially; anterior paracoxal suture of mesothorax complete; furcasternal protuberances of mesosternum not contiguous, separated by a median impression widened posteriorly; *Wings* (**iii**) MP fork of forewing situated distally, and branched after 0.25 of its length; MP fork of hind wing situated proximally, and branched after 0.19 of its length; *Legs* (**iv**) first tarsomere of hind leg fused with tibia, comprising app. 1/4 of its length; pretarsal claws of all legs dissimilar, with outer claw hooked and inner claw blunt.

The new extinct species described here differs well from all other representatives of Ameletopsidae by its small body size and wings. Extant genera of Ameletopsidae are considerably larger, sometimes more than twice as large as *Nebesna* **gen. nov.** (Supplement Table 1). Although the male imago of *Nebesna* **gen. nov.** is incompletely preserved (part of abdominal segment IX, segment X, genitalia and cerci are missing), we can assume with certainty that the total body length did not exceed 7.5 mm. Within the discussed extant and extinct lineages of Southern Hemisphere Siphlonuroidea, only Mesozoic Astraeopteridae are similar in body and wing size, but clearly differ in the structure of thorax^2^.

The absence of male genitalia, cerci and terminal filament of the new fossil genus somewhat complicates the analysis of its systematic relationships. Also, adult body and wing colouration should be excluded from the analysis, as in recent species of Ameletopsidae it can vary from dark brown to lemon yellow, with wings hyaline and covered with dark patches, to more or less uniformly light yellow. The coloration of mayfly specimens embedded in fossil resins is mainly uniform light yellow to dark brown or blackish and very rarely corresponds with the natural colouration of the living insect. Only in a few adult specimens described earlier, the traces of natural pigmentation are preserved on different body parts (mostly on thorax). The male subimago of *Electroletus soldani* Godunko & Neumann, 2006 described from the Eocene Baltic amber was attributed to the family Ameletidae based on preserved traces of pigmented area on submedioscutum and sublateroscutum of mesonotum, a character listed within the apomorphies of this family (pp. 80–82, figs. 21B in^16^; pp. 178–179, fig. 9 in^48^). Similar traces of mesonotum pigmentation are described for a single specimen of the monospecific genus and family Babidae Kluge et al. 2006 (Ephemeroidea) also described from this type of the fossil resin (see pp. 182–185, fig. 6 in^49^).

Besides the mentioned distinct differences in body and wing size, we can distinguish *Nebesna* **gen. nov.** also based on the following features:

(i) Male compound eyes well developed in all representatives of Ameletopsidae, however only in *Nebesna* **gen. nov.** lower portion of the eye is clearly short, approximately 0.62 × as long as the upper portion (Fig. 1C, E; Supplementary Table S1).
(ii) Basitornal margin of the forewings conspicuously shortened, slightly more than half the length of the tornoapical margin (in contrast to markedly elongated basitornal margin in *Chiloporter* and *Mirawara*); basitornal margin of forewing is clearly elongated also in recent genera *Chaquihua*, *Ameletopsis,* and fossil *Balticophlebia* (Fig. 7C). As a result, forewing tornus is shifted more proximally in *Nebesna* **gen. nov.**, in contrast to a tornus situated more distally in the remaining Ameletopsidae. Several differences can be also found in the forewing venation, namely in the position of MA and MP furcation, and the position of the proximal end of iRS. The cubital field is relatively short and narrow in *N. sotnia* **gen. & sp. nov.** in comparison to the long and wider cubital field in other Ameletopsidae. The importance of this character in the context of the investigation of amphinotic mayflies was pointed out by Philips^14^, who schematically depicted the different wing shapes in siphlonuroid taxa and the cubital fields bordered by CuA and CuP. In addition, *Nebesna* **gen. nov.** has the smallest number of stout cubital intercalaries within Ameletopsidae, all of these veins are simple without any branching or small intercalaries between each other. While the distal ends of CuP and A_1_ in the forewings of other Ameletopsidae are not approximated or only slightly approximated, in *Nebesna* **gen. nov.** the apical part of both veins is clearly approximated (Fig. 3A–C; Supplementary Table S1).
(iii) The hind wing of the new genus is relatively narrow and moderately rounded distally, in contrast to the broadly shaped wings in *Balticophlebia* (Eocene) and *Chiloporter* (extant, S. America). The shape of hind wings in other genera of Ameletopsidae is more or less broad, with widely rounded distal end. The prominent costal process is apically pointed in *Nebesna* **gen. nov.**, in opposite of the broadly rounded costal process in the remaining Ameletopsidae. While the tornoapical margin of *Nebesna* **gen. nov.** and *Chaquihua* have no traces of a fold, other Ameletopsidae are equipped with a visible fold situated mainly near MP field, occasionally in MA or between MP and CuA (see fig. 9 in^10^; fig. 2a in^35^; fig. 2a in^50^). The positions of MA–RSp fork and MP fork also differ in *Nebesna* **gen. nov.** Finally, the cross venation in all wings is poorly developed, and the number of basally connected intercalaries in all vein fields of both wing pairs is the smallest of all the Ameletopsidae (Fig. 4A–C; Supplementary Table S1).

### Palaeoecological considerations

The extant species of different Siphlonuroidea lineages inhabit several types of water courses, from fast cold-water streams to broad, flat, slow-flowing rivers. Thus, their larvae are found in a wide range of ecological conditions, on rocky bottom substrates, littoral vegetation, or at different depths in sand-bottomed rivers^37,51–53^. Shredders feeding on coarse particulate organic matter are most commonly occurring among Siphlonuroidea, and their mouthparts are not particularly specialised. Exemptions are the obligatorily carnivorous larvae of Ameletopsidae and Acathametopodidae with respectively specialised mouthparts^16,51,54,55^. On the other hand, the considerable diversity of genera with recent representatives known on larvae provides grounds to assume that the range of hydrological characteristics of paleo-rivers in the discussed period and region was exceptionally wide, and included both rapidly flowing streams with numerous branches and cold-water pools, as well as foothill and plain rivers with finely sedimented substrate of the riverbed, from small pebbles to sand.

### Biogeographical considerations

Considering the historical changes in ecosystems at the biome level as a response to global climatic fluctuations under the impact of geological and extraterrestrial collisions, different evolutionary and adaptive potentials of particular taxa, a grounded reconstruction of biogeographic relationships could only be assured by analysing both recent and fossil taxa. The most successful seems to be an attempt to interpret data on geological processes related to the migration of land parts, overlaid on time-calibrated phylogeny resulting both morphological and molecular dataset, using calibration fossils. This phylogeographical approach is still uncommon for mayflies^56–58^, although it has yielded important data for other animal groups^59–64^.

*Paleontological data.* The new discovery of a male imago from the Eocene of Europe, assigned here to the family Ameletopsidae, will not only be useful for dating this clade, but also radically change our understanding of its distribution in space and time. While for some large mayfly families such as Leptophlebiidae, Baetidae, Caenidae, and possibly Polymitarcyidae, a Pangean origin can be inferred with high probability^26,33,65,66^, the spatial and temporal reconstructions for small families is a more challenging issue. In these cases, we are dealing with the presence of a small number of recent taxa within respective families, occurring rather compactly, not extending beyond one of the biogeographic realms. On the other hand, there is very limited or even no fossil evidence of such small families, which distinctly limits the analysis of its spatial and temporal dynamics.

In order to reconstruct the evolutionary and spatial trends of these families, the presence of fossil evidence that shed light on historical ranges is significant. For example, fossils of the family Baetiscidae, which is solely distributed in the Nearctic nowadays, were first of all discovered in Eocene Baltic amber (for more details see^66^), and its spatio-temporal pattern resembled that of *Analetris* (Acathametropodidae) or *Siphloplecton* (Metretopodidae). Later, however, larval fossil remains of this family were identified from the Early Cretaceous Crato Formation of Brazil, and Early Cretaceous Koonwarra Fossil Bed of Australia (Fig. 7E, F; see also^28,33,66^). This provided the possibility, following Pescador et al.^67^, to finally confirm a Pangean distribution of the family Baetiscidae. The family Coloburiscidae Landa, 1973 was suggested as probably taxa with Gondwanan origin by Barber-James et al.^26^. Nevertheless, numerous records of adult specimens of the genus *Cronicus* Eaton, 1871 in Baltic amber, which was assigned to Coloburiscidae (Fig. 7G, H; see also^35^), may also indicate the ancient nature of this family.

The current distribution of Ameletopsidae includes Australia (genus *Mirawara*), New Zealand (*Ameletopsis*), and South America with Chile and Argentina (*Chaquihua* and *Chiloporter*). Moreover, there is no doubt that this family has existed in Australia for at least the last 100 My. The peculiar Cretaceous larvae of the genus *Promirara* from the Koonwarra Fossil Bed described by Jell & Duncan^28^ undoubtedly belong to Ameletopsidae (Fig. 7A, B; see also^16^). Therefore, Barber-James et al.^26^ assumed Gondwanan origin for Ameletopsidae, despite the fact that female imago of *B. hennigi* was described from Baltic amber and initially had been attributed to this family^35^. Although Kluge^16^ placed the genus *Balticophlebia* to Anteritorna *incertae sedis*, *B. hennigi* shares the key diagnostic characters of the remaining Ameletopsidae, namely features of fore- and hind wing venation, posteriorly separated mesofurcasternal protuberances), a strongly reduced terminal filament (to 10 segments), and the presence of posterolateral projections on abdominal segment IX (Fig. 7C; Supplementary Table S1).

With the description of *Nebesna sotnia* **sp. nov.** and the presence of the still undescribed female imago of Ameletopsidae in Miocene Dominican amber (Fig. 7D; Staniczek & Godunko, *in preparation*), additional puzzle pieces in the Pangaean pattern of this family could be discovered.

*Phylogenetic data.* In contrast to Kluge et al.^1^, who suggested monophyly for the family group of Southern Hemisphere Siphlonuroidea, Ogden et al.^39^ not only assumed Siphlonuroidea as paraphyletic, but initially even assumed Ameletopsidae to be paraphyletic due to their molecular data set. More recently, Ethington & Ogden^68^ reanalysed a molecular subset of the taxa from Ogden et al.^39^, which again rendered Ameletopsidae as paraphyletic, but the authors suggested a high probability of error in extraction or coding. The combined phylogenetic analysis based on morphological and molecular data presented by Ogden et al.^69^ rendered Ameletopsidae as monophyletic group, and sister to Rallidentidae + Siphlaenigmatidae + Oniscigastridae. At the same time, Nesameletidae are indicated as sister to Ameletidae + Siphlonuridae.

Gonser^70^ considered that phylogenetic relationships within the families Ameletopsidae, Oniscigastridae, and Nesameletidae (and apparently Rallidentidae) reflect the sequence of geological events that occurred in the eastern part of Gondwana. That is, New Zealand was the first to separate around 85–83 My ago^71,72^, and Australia and Antarctica followed starting to separate in the Jurassic around 160 My^73^, but remained connected in one way or another until the Eocene, when the final link between them was broken by the South Tasman Rise, which occurred from around 35–32 My^74,75^ or up to 45 My according to van den Ende et al.^76^. Finally, the last terrestrial connection between Antarctica and South America through the Antarctic Peninsula was consistently broken during the final stage of the Gondwana breakup (about 80 My), with the complete separation occurring around 40 My^63,76–78^.

Kluge et al.^1^ suggested that Siphlonuroidea may be of Northern Hemisphere (Laurasian) origin, and one of their ancient representatives crossed into Gondwana and distributed there. The isolation of the four amphinotic families (Oniscigastridae, Nesameletidae, Ameletopsidae and Rallidentidae), according to Kluge et al.^1^, must have occurred before the separation of South America, Antarctica, Australia and New Zealand; the absence of these families in other parts of Gondwana (Africa, India and Madagascar), according to these authors, indicates their shaping after the breakdown of the connections between these land masses. On the other hand, given the stenobiontic character of amphinotic larvae, which are often restricted to cold water streams, Kluge et al.^1^ also considered an alternative scenario according to which these families formed before the separation of Africa, India, and Madagascar from Gondwana, where they then became extinct as a result of climatic changes.

Both Ameletopsidae and Nesameletidae share a similar distribution of phylogenetic lineages identified by Ogden et al.^69^ in their latest reconstruction. In Ameletopsidae, the Australian genus *Mirawara* is sister to *Ameletopsis* [New Zealand] + *Chaquihua* [S. America], while in Nesameletidae in a similar way, *Ameletoides* [Australia] is sister to *Nesameletus* [NZ] + *Metamonius* [SA]. The sister group of Ameletopsidae is Rallidentidae + Siphlaenigmatidae + Oniscigastridae, of which both monogeneric *Rallidens* and *Siphlaenigma* are distributed exclusively in New Zealand. In Oniscigastridae, *Oniscigaster* [NZ] is revealed as sister to *Tasmanophlebia* [AUS] + *Siphlonella* [SA]^69^.

Considering all available data, the historical distributional pattern of the family Ameletopsidae at the current state of knowledge may be suggested under the following possible scenario:

**(i)** Ameletopsidae may have originated as a distinct family not later than the beginning of the Early Jurassic (ca. 200 My), from an ancestor that may have been one of the first crowngroup taxa in the Late Triassic. Pescador et al.^67^ reached a similar conclusion for the Baetiscidae with a similar historical distributional pattern, suggesting an age of at least 200 My (see also^33^). For Plecoptera, earlier ages for major divergences were justified by Cui et al.^61,79^ and Sroka & Prokop^80^, assigned these to the Early Mesozoic or Late Paleozoic.

A Pangaean origin of Ameletopsidae would suggest their rapid diversification and dispersal southward, even before the breakup of the continents of Gondwana and Laurasia (215–175 My). At least some of the early lineages of Ameletopsidae remained in Laurasia, ancestral to the future genera *Balticophlebia* and *Nebesna* **gen. nov.**, which existed in Europe until Lutetian stage of the Eocene (34–48 My).

**(ii)** Due to a rapid dispersal in a southern and southwestern direction, one of the Ameletopsidae clades reached North America in the Late Toarcian age at latest (approximately 175 My). However, dispersal could have occurred in the opposite direction, accompanied by the gradual separation of Eastern and Western Laurasia. Nevertheless, the breakup of Laurasia was not a vicariant key event in the Jurassic and Cretaceous for many Siphlonuroid families. The faunal similarities at generic level between the two former Laurasian parts are well documented in Eocene Baltic amber (see above), not only in Ephemeroptera, but also in other taxa of aquatic insects. Their areas were large, covering North America and Europe (see^81^ for Plecoptera;^82^ for Trichoptera;^83^ for Diptera). Such dispersal may have occurred along one of the three described corridors or routes, namely the Bering Land Bridges, Thulean route, and De Geer route.

In one way or another, Ameletopsidae survived in the former Eastern Laurasia until the Eocene and in its Western part until the Miocene. Despite the significant geological fluctuations accompanying the southern part of North America, Central America and the Caribbean in particular, there have always existed large and stable land masses with mountain ranges from the Early Jurassic on that were able to provide specific environments for a diverse fauna of rheophilic invertebrates^84–87^. A Miocene species of Ameletopsidae, recently discovered by us in Dominican amber, like other elements of the mayfly fauna may have colonized the Caribbean islands before or during the Miocene, mainly from the mountain ranges of Central America or northern part of South America.

**(iii)** The ancestor of the recent Ameletopsidae most probably originated from the “Western Laurasian” branch. Therefore, the “alternative” version of the origin and age of this family proposed by Kluge et al.^1^ could be very probable: Ameletopsidae migrated southward in the Middle Jurassic through Africa, India and Madagascar, where they became extinct due to climatic changes and the loss of appropriate ecological niches, as well as geological events associated with the drift of these land masses. Unfortunately, paleontological records of mayflies from these regions are sparse and belong to other superfamilies^88,89^.

**(iv)** After reaching Eastern Gondwana no later than 160–150 My ago, Ameletopsidae diversified rapidly there during the Late Jurassic and Early Cretaceous, spreading throughout its whole territory. This is supported by the larvae of the Ameletopsid genus *Promirara* from the Early Cretaceous Koonwarra Fossil Bed (118–115 My)^28,33^.

Together with Ameletopsidae, other amphinotic families such as Nesameletidae, Oniscigastridae and Coloburiscidae also include vicariant genera restricted to Australia, New Zealand, and the southern part of South America^26,90–92^. Besides Siphlonuroidea, similar spatial and temporal patterns can also be observed in Leptophlebiidae: Atalophlebiinae (for more details and bibliography see^58^). Thus, the interchange of faunas between these regions using an East Gondwanan corridor indicates that there was once an important centre of evolution in the Southern Hemisphere, with a significant role of Antarctica^26^. The paleoclimatic conditions of Antarctica, based on soil and vegetation studies, at the end of the Lower Cretaceous (Albian age; about 113–100 My) included a warm and humid climate with the presence of permanent rivers^93^. In the Oligocene, the climate changed significantly and became distinctly cooler.

**(v)** The breakup of New Zealand and remaining Gondwana followed by the Australian–Antarctic breakup in the Late Cretaceous were important vicariant events that fragmented the habitats of the amphinotic taxa. Based on the phylogenetic analysis^69^, it can be assumed that South America, Antarctica and New Zealand shared a similar, diverse siphlonuroid fauna before the separation of these land masses, while an easternmost clade became isolated in Australia. The phylogenetic splitting of Ameletopsidae on *Mirawara* and *Ameletopsis* + *Chiloporter–Chaquihua* may have occurred at least in the beginning of the Lower Cretaceous (no later than 150–145 My), but long before the separation of New Zealand, providing sufficient time for the evolution of a diverse East Gondwanan fauna.

Using the Antarctic corridor through the Antarctic Peninsula to the southern part of South America, the ancestor of the *Chiloporter* + *Chaquihua* lineage migrated until the Eocene–Oligocene border or earlier (37–30 My), when climatic conditions and tectonic situation still enabled these transitions^63,94^.

Several studies indicate that amphinotic mayflies and other insect groups show a pattern, where the South American taxa are closer related to the Australian taxa than to those of New Zealand^58,91,95–100^. However, with high probability Ameletopsidae and Nesameletidae show a different phylogenetic pattern with closer relationships of New Zealand and South American taxa^69^.

**(vi)** Further events in the distribution and evolution of amphinotic mayfly families in South America are obviously closely related to a second pulse of uplift and eastward migration of the orogenic front occurred during the Early-Middle Miocene in the Andean Cordillera^101^. The current distribution of these families is restricted between 33 and 55 parallels of south latitude, within the South-Central and South Andes, the marginal foothills of Patagonia, and Tierra del Fuego in Chile and Argentina^6–8,21–23,25,102^. However, in the past, the area of these families may have extended along the Andean Mountain range chain, because the first phases of Andean uplift in the Early Cenozoic did not represent a barrier to faunal exchange^102^. After the ancestors of amphinotic families from Antarctica had migrated to the south of South America (presumably Eocene), there was sufficient time for their dispersal in the cold-water rivers and streams of the Cordilleras. Additionally, the Eocene seems to have been a relatively “calm” period with few disturbing geologic events in this region, which may have contributed to the rapid dispersal of aquatic invertebrates.

The second phase of orogeny during the Miocene-Pliocene–Quaternary interval is characterized by a rapid rise within the Andean Mountain system, with extensive tectonic processes that certainly had a significant impact on the distribution of recent insect groups, therefore becoming an important vicariant event in South America^101–103^. About 11–15 My (Middle Miocene), the Andes uplifted to about 3000 m a.s.l.^101,104^, an altitude sufficient to provide such vicariant barrier. The orogeny may have pushed back southward the former Gondwanan migrants from the Northern and Central Andes, where they became extinct. Eastward migration of cold-water rheophilic fauna was limited by the natural barrier of the Patagonian highlands, plains, and desert. Thus, the current distribution of amphinotic families in South America reflects sequential processes where vicariance, dispersal and extinction events have played a role in their history.

An alternative scenario would be the Gondwanan origin of Ameletopsidae, as well as other amphinotic families. This would also explain the presence of *Promirara* in the Cretaceous of Australia, as well as the recent distribution of Ameletopsidae. The family would have originated in Gondwana, spreading later to Europe and North America (Caribbean region) by using one of the paths through Africa or South America, and then become extinct in Northern Hemisphere in Cenozoic. However, this may be contradicted by modern data on tectonic events throughout the Jurassic, and especially in the Cretaceous, when transcontinental jumps northwards for possibly stenobiont cold-water predators such as Ameletopsidae were most likely impossible. The latest phylogenetic reconstruction of Ephemeroptera^69^ indicates regarding to the Siphlonuroid families that the Northern families Siphlonuridae + Ameletidae are sister to the Southern family Nesameletidae, which altogether are sister to the remaining Southern Siphlonuroid families. In turn, this entire clade is sister to the Metretopodidae and Heptagenioidea McCafferty, 1997 (including Isonychiidae Landa, 1973) from the Northern Hemisphere. This may also point to an older history of the Ameletopsidae in the Northern Hemisphere than previously assumed.

## Material and methods

### Material

The piece of Baltic amber including *Nebesna sotnia* **sp. nov.** originates from the Yantarny amber mine (formerly Palmnicken), Sambia (Samland) Peninsula, Kaliningrad Region, Russian Federation.

The holotype is currently deposited in the collection of the National Museum of Natural History, National Academy of Sciences of Ukraine, under inventory number IKOFZ-Ib 8921, and available to researchers upon reasonable request.

Comparative adult and larval material of fossil Ameletopsidae are housed in the Geowissenschaftliches Zentrum, Universität Göttingen, Germany [holotype of *Balticophlebia hennigi* Demoulin, 1968, female imago, reg. no. 36, Eocene Baltic amber, originates from Kaliningrad Region in Russian Federation; Fig. 7C], the State Museum of Natural History, Stuttgart, Germany [undescribed, presumably new species and genus of Ameletopsidae, female imago, Do-1268-K, Miocene Dominican amber, Dominican Republic; Fig. 7D], and the Victoria Museum, Melbourne, Australia [holotype and paratypes of *Promirara cephalota* Jell & Duncan, 1986, larvae, originates from the Early Cretaceous Koonwarra Fossil Bed in Australia; Fig. 7A, B].

### Optical equipment, measurements, terminology

Serial photographs of the holotype of *Nebesna sotnia* **gen. & sp. nov.** in different focal planes were taken with a Canon 90D and Canon MP-E-65 lens. The holotype of *B. hennigi* and undescribed female imago of Ameletopsidae from Miocene Dominican amber was photographed through a Leica Z16 APO Macroscope equipped with a Leica DFC450 Digital Camera using Leica Application Suite v. 3.1.8. Resulting photo stacks were processed with Helicon Focus Pro 6.4.1 and Helicon Remote (Kharkiv, Ukraine) to obtain combined photographs with extended depth of field. The same optical equipment and photo-technic were used for investigation of the comparative adult material of the extant representatives of Ameletopsidae, Nesameletidae, Oniscigastridae, and Rallidentidae housed in ENTU and SMNS collections (Supplementary Table S1).

The measurements of individual body parts were inferred from photographs taken with a calibration scale or ocular grid (Supplementary Table S1). The descriptive morphological terminology follows^16,40,105–107^; for systematic classification we followed Kluge et al.^1^ and Kluge^16^.

### Nomenclatural acts

The electronic edition of this article conforms to the requirements of the amended International Code of Zoological Nomenclature, and hence the new names contained herein are available under that Code from the electronic edition of this article. This published work and the nomenclatural acts it contains have been registered in ZooBank, the online registration system for the ICZN.

The ZooBank LSID for this publication is: urn:lsid:zoobank.org:pub:14D53A7F-C483-4A12-B973-EAFD6A5B282E.

## Conclusion

The new monospecific genus *Nebesna sotnia* **gen. & sp. nov.** described from Eocene Baltic amber is a new representative of a previously unknown diverse fossil fauna systematised within the basal paraphyletic superfamily Siphlonuroidea. The new genus is characterized by a complex of imaginal diagnostic characters allowing its attribution to the family Ameletopsidae. This family is of amphinotic distribution and represented by extant genera and species in Australia, New Zealand and South America. Complementing the earlier fossil record of Ameletopsidae from the Early Cretaceous of Australia, the presence of *Nebesna* **gen. nov.** in the Eocene of Europe suggests an older history of this family, which dates well back before the breakup of Pangea. We describe the historical events linked to the evolutionary distributional pattern of Ameletopsidae as a sequential process, in which vicariance, dispersal, and extinction played an important role. Supporting previous suggestions about the earlier age of diversification of major phylogenetic lineages in mayflies and stoneflies, we attribute the earliest age of this family to the Early Jurassic, prior to the time of the final breakup of Pangea. Considering the discovery of yet another fossil representative of Ameletopsidae in Miocene Dominican amber, we hypothesize a Jurassic dispersion of the ancestral Ameletopsidae into Western Laurasia in the Late Toarcian age, with a simultaneous expansion southward, possibly through Africa, Madagascar, and India, until reaching Eastern Gondwana no later than 160–150 My ago. Here, Ameletopsidae must have dispersed over the entire landmass. Phylogeny grounded on genetic analysis shows that in Ameletopsidae (as well as in some other amphinotic families) the Australian clade is sister to a branch composed of New Zealand and South American genera. This could support the assumption that all major clades of amphinotic families were already developed in the Early Cretaceous, well before the split off of Gondwana, with a distribution over a wide area of the Eastern Gondwana, including Antarctica, which was an important centre of evolution in the Southern Hemisphere. Finally, through the Antarctic Peninsula, as long as paleotectonic and climatic situation allowed, Ameletopsidae would have reached South America no later than the Eocene, where they are currently preserved in a relatively small and narrow area of the mountain range of the Southern Andes.

## Supplementary Information

Supplementary Information 1.

Supplementary Information 2.

## Data Availability

All data generated or analysed during this study are included in this published article. All relevant data are available from the authors. The datasets generated during and/or analysed during the current study are available from the corresponding author upon reasonable request. One fossil specimen embedded in the Eocene Baltic amber used in this study is housed in the National Museum of Natural History (Kyiv, Ukraine). As one of the co-authors of current publication, Jonas Damzen, donated the holotype for NMNH NASU (Kyiv). Available inventory number of the holotype (i.e. IKOFZ-Ib 8921) is listed in this published article. Requests for access to the fossil material should be addressed to the curator of the collection and corresponding author of this article. This work has been registered online at zoobank.org under LSID urn:lsid:zoobank.org:pub:14D53A7F-C483-4A12-B973-EAFD6A5B282E. The new taxa are registered in Zoobank.org.
