# Supplementary material for: Nebesna sotnia gen. & sp. nov. from Baltic amber supports a Pangean distribution of the amphinotic family Ameletopsidae (Ephemeroptera)
Source: Sci Rep. 2025 May 26;15:18415. doi: 10.1038/s41598-025-01722-8 (PMC12106746; doi:10.1038/s41598-025-01722-8)
Supplement: Supplementary file 1 — Supplementary Information 1. [file 41598_2025_1722_MOESM1_ESM.docx]

**Supplementary Table S1.** Summary of adult characters of extant and extinct Siphlonuroidea of the Southern Hemisphere, with focus on the representatives of the family Ameletopsidae (including fossil genera from Cenozoic of Europe).

| **Characters** | **Ameletopsidae Edmunds, 1957** | | | | | | | | | | | **†AST** | **NES** | **ONI** | **RAL** |
| --- | --- | --- | --- | --- | --- | --- | --- | --- | --- | --- | --- | --- | --- | --- | --- |
|  | **CHA** | **CHI** | **MIR** | | **AME** | | **†BAL** | | **†NEB** | | |  |  |  |  |
|  | **Extant**  **[Chile,**  **Argentina]** | **Extant**  **[Chile,**  **Argentina]** | **Extant**  **[Australia]** | | **Extant**  **[New Zealand]** | | **Eocene**  **Lutetian**  **34–48 Ma**  **[Europe]** | | **Eocene**  **Lutetian**  **34–48 Ma**  **[Europe]** | | | **Lower Cretaceous**  **Aptian**  **app. 113 Ma**  **[Brazil]** | **Extant**  **[Australia, New Zealand,**  **Chile,**  **Argentina]** | **Extant**  **[Australia, New Zealand,**  **Chile,**  **Argentina]** | **Extant**  **[New Zealand]** |
|  | **male / female** | **male / female** | **male / female** | | **male / female** | | **female imago** | | **male imago** | | | **adults** | **male / female** | **male / female** | **male / female** |
| ***Measurements*** | | | | | | | | | | | | | | | |
| Body length [mm] | 10.00–15.50 / 12.00–18.00 | 16.00–19.00 / 15.50–22.00 | 15.20–17.50 / 16.00–18.00 | | 14.00–16.00 / 15.00–18.00 | | 15.00 | | **7.28*** | | | **7.60*–13.20*** | 10.00–16.50 / 9.00–18.00 | 18.00–20.00 / 20.00–24.00 | 10.00–15.30 / 13.90–16.10 |
| Forewing length [mm] | 16.50–18.00 / 20.00–21.00 | 17.00–17.50 / 19.50–21.00 | 17.00–18.00 / 17.00–20.00 | | 17.00–18.00 | | 16.00 | | **7.80** | | | **7.65*–11.40** | 11.00–18.00 / 10.00–20.00 | 18.00–20.00 / 19.00–21.00 | 10.50–13.80 / 13.50–16.80 |
| Forewing width [mm] | 6.20–8.00 | 5.60–7.00 | 5.40–5.96 | | 6.80–7.10 | | 6.40 | | **2.65** | | | **3.62*–6.30** | 2.90–3.30 | 5.20–5.60 | 4.00–5.40 |
| Forewing  [width / length ratio] | 0.36–0.39 | 0.32–0.35 | 0.30–0.34 | | 0.38–0.40 | | 0.40 | | 0.34 | | | 0.47–0.55 | 0.23–0.29 | 0.27–0.29 | 0.38–0.44 |
| Forewing [basitornal / tornoapical margin length ratio] | 0.66–0.70 | **0.82–0.84** | 0.72–0.76 | | 0.62–0.65 | | 0.66 | | 0.56 | | | 0.54–0.64 | 0.55–0.70 | **0.74–0.82** | **0.75–0.80** |
| Tornus [location relative to forewing length] | **0.36–0.39** | 0.42–0.47 | 0.42–0.46 | | 0.38–0.40 | | 0.40 | | **0.36** | | | 0.30–0.34* | 0.35–0.45 | 0.45–0.50 | 0.40–0.42 |
| Hind wing length [mm] | 6.80–7.20 | 8.00–9.40 | 7.40–9.20 | | 7.40–7.80 | | 5.90 | | **2.63** | | | **≈2.00–3.72** | 4.00–8.00 | 7.60–7.80 | 4.90–6.50 |
| Hind wing width [mm] | 3.50–4.00 | 4.60–5.20 | 4.30–4.50 | | 4.00–4.25 | | 3.46 | | 1.38 | | | – | 2.20–4.40 | 4.20–4.40 | 3,20–4.36 |
| Hind wing  [width/length ratio] | 0.52–0.55 | 0.55–0.58 | 0.50–0.58 | | 0.50–0.52 | | 0.56 | | 0.52 | | | – | 0.52–0.58 | 0.52–0.56 | 0.65–0.67 |
| Hind / forewings length ratio | 0.35–0.39 | **0.45–0.47** | **0.43–0.50** | | 0.40–0.42 | | 0.37 | | 0.40 | | | ≈0.18–0.45 | 0.35–0.40 | 0.42–0.45 | 0.40–0.43 |
| ***Head* (Figs. 1, 2)** | | | | | | | | | | | | | | | |
| Compound eyes of male [shape] | large | large | large | | large | | – | | large | | | – | large | large | large |
| Compound eyes of male [lower/upper portion max. length ratio] | 0.76–0.80 | 0.70–0.74 | 0.84–0.90 | | 0.92 | | – | | **0.62** | | | – | 0.90–1.00 | 0.80–0.90 | 0.80 |
| Upper portion of male compound eyes [structure] | contiguous medially | contiguous medially | contiguous medially | | nearly contiguous | | – | | contiguous medially | | | – | **separated medially** | **separated medially** | contiguous medially |
| Medial projection of vertex | absent | absent | absent | | absent | | absent | | absent | | | absent | **present,**  **unpaired** | absent | absent |
| ***Thorax* (Figs. 1, 2, 5, 6)** | | | | | | | | | | | | | | | |
| *Thorax* [shape] | moderately elevated above head | moderately elevated above head | moderately elevated above head | | moderately elevated above head | | moderately elevated above head | | moderately elevated above head | | | **markedly elevated above head** | moderately elevated above head | moderately elevated above head | moderately elevated above head |
| *Mesothorax* | | | | | | | | | | | | | | | |
| Mesonotal suture [MNs; shape] | nearly transverse | nearly transverse or shortly stretched backward medially | | nearly transverse | | nearly transverse | | nearly transverse | | shortly stretched backward medially | | nearly transverse | nearly transverse or shortly stretched backward medially | nearly transverse or zigzag shaped | nearly transverse |
| Lateroparapsidal suture [LPs; shape] | relatively short, straight distally | relatively short, straight distally | | relatively short, straight distally | | relatively short, straight distally | | moderately elongated, slightly bent inward | | relatively short, straight distally | | ? short, bent inward distally | relatively short, straight or slightly bent outward distally | relatively short, slightly bent inward distally | relatively short, straight distally |
| Anterior paracoxal suture [PCxsA; structure]** | complete | complete | | complete | | complete | | complete | | complete | | complete | complete | complete | **incomplete** |
| Furcasternal protuberances [FSp; shape] | not contiguous | not contiguous | | not contiguous | | not contiguous | | not contiguous | | not contiguous | | ? not contiguous | not contiguous | not contiguous | not contiguous |
| Furcasternal median impression [shape] | narrow anteriorly, widened posteriorly | narrow anteriorly, widened posteriorly | | narrow anteriorly, widened posteriorly | | narrow anteriorly, widened posteriorly | | parallel-sided, not widened posteriorly | | **narrow anteriorly, strongly widened posteriorly** | | – | narrow anteriorly, widened posteriorly | parallel-sided, not widened posteriorly | parallel-sided, not widened posteriorly |
| ***Forewing* (Figs. 1A, B, 3, 5E, 6E)** | | | | | | | | | | | | | | | |
| Costal brace | moderately arched | moderately arched | | strongly arched | | strongly arched | | strongly arched | | | strongly arched | arched | arched or sloping | arched or sloping | moderately arched |
| Pterostigma [number of cross veins] | 12–15 | 13–20 | | 14–18 | | 15–20 | | more than 16 | | | app. 13 | – | 9–18 | 10–18 | up to 16 |
| Pterostigma [shape of veins] | mostly forked | mostly simple | | mostly simple | | simple and forked | | simple | | | simple and forked | – | simple and forked | simple and forked | mainly forked |
| RP fork [place of furcation] | 0.20–0.22 | 0.22–0.25 | | 0.16–0.20 | | 0.15–0.17 | | 0.16 | | | 0.15 | 0.15–0.23 | 0.20–0.28 | 0.10–0.15 | 0.26–0.27 |
| RSa_2_’’ [shape] | long, arises from RSa_1_ | free, long | | free, long | | long, connected to RSa_1_ and iRS basally | | free, long | | | free, short | free, short | long, arises from RSa_1_ | long, connected to RSa_1_ and iRS basally | free, short |
| iRS [basally] | free, long, approximated to RSa_1_ | free,  approximated to RSp | | free | | free | | free | | | **arises from RSa_1_** | free, approximated to RSa_1_ | free | free | free, slightly approximated to RSa_1_ |
| MA fork [shape] | slightly asymmetrical | nearly symmetrical | | slightly asymmetrical | | nearly symmetrical | | slightly asymmetrical | | | nearly symmetrical | nearly symmetrical / slightly asymmetrical | symmetrical or nearly symmetrical | slightly asymmetrical | slightly asymmetrical |
| MA fork [place of furcation] | 0.52–0.55 | 0.43–0.45 | | 0.46–0.48 | | 0.50–0.52 | | 0.50 | | | 0.45 | 0.46–0.65 | 0.50–0.56 | 0.50–0.55 | 0.50–0.52 |
| MP fork [shape] | asymmetrical | asymmetrical | | asymmetrical | | asymmetrical | | asymmetrical | | | asymmetrical | asymmetrical | asymmetrical | asymmetrical | asymmetrical |
| MP fork [place of furcation] | 0.13–0.15 | 0.12–0.14 | | 0.10–0.13 | | 0.18–0.20 | | 0.20 | | | **0.25** | 0.07–0.13 | 0.10–0.20 | 0.15–0.20 | 0.14–0.16 |
| MP_2_ [basal connection] | to MP_1_ | **to iMP or MP_1_** | | to iMP | | to MP_1_ | | to MP_1_ | | | to MP_1_ | to MP_1_ | **to MP_1_ or CuA** | to MP_1_ | to MP_1_ |
| iMP [length respective to MP_2_] | shorter | **longer or shorter** | | longer | | shorter | | shorter | | | shorter | shorter | shorter | shorter | shorter |
| iMP [proximal end] | free  [connected basally to MP_1_ and MP_2_] | **free**  **[connected basally to MP_1_ and CuA or MP_2_]** | | free  [connected basally to MP_1_ and MP_2_] | | free  [connected basally to MP_1_ and MP_2_] | | free  [connected basally to MP_1_ and MP_2_] | | | free  [connected basally to MP_1_ and MP_2_] | approximated to MP_1_ | **free or arising from MP_1_, or arising from MP_2_** | **arising from MP_1_** | free  [connected basally to MP_1_ and MP_2_] |
| Cubital field [shape] | long and narrow | long and narrow | | long and narrow | | long and narrow | | not narrow | | | **relatively short and narrow** | long and narrow | long and narrow | long and narrow | long and narrow |
| Cubital stout intercalaries [iCu; number of veins] | 5–7 | 6–8 | | 7–8 | | 7–8 | | 5 | | | **4** | 4–8 | 5–10 | 5–9 | 5–6 |
| Cubital stout intercalaries [iCu; shape of veins] | mainly simple | mainly simple | | simple, occasionally forked | | simple | | simple | | | simple | simple | simple, occasionally forked | simple, occasionally forked | simple and forked |
| Cubital field [small basally free intercalaries] | present | **absent** | | **absent** | | present | | present | | | **absent** | present | present | present | present |
| CuP [distal end] | slightly bent | not bent or slightly bent | | slightly bent | | slightly bent | | not bent | | | slightly bent | not bent | bent or sharply bent | slightly bent | sharply bent |
| CuP and A_1_ [distal end] | not approximated | moderately approximated | | not approximated | | not approximated | | not approximated | | | **approxima-ted** | not approximated | **approxima-ted** | not approximated | not approximated |
| Anal field [longitudinal veins] | poorly developed | moderately developed | | well developed | | poorly developed | | poorly developed | | | poorly developed | poorly developed | rich, well developed | well developed | poorly developed |
| Anal field [cross veins] | mainly absent | present | | mainly absent | | mainly absent | | – | | | absent | – | mainly absent | mainly absent | absent |
| A_1_ [number of additional veins going to basitornal margin] | 2–3 | 2–3 | | 1–2 | | 1–2 | | – | | | – | – | 1–3 | 1–2 | – |
| Cross venation of fore- and hind wings | rich, well developed | rich, well developed | | rich, well developed | | rich, well developed | | rich, well developed | | | **moderately developed** | – | well developed | well developed | well developed |
| Basally connected intercalaries [along tornoapical margin of wing] | rich, well developed | rich, well developed | | rich, well developed | | rich, well developed | | rich, well developed | | | **not numerous** | – | rich, well developed | rich, well developed | well developed |
| Wing pigmentation [in imago] | present | present | | present | | present | | **absent** | | | **absent** | – | present or absent | present | **absent** |
| ***Hind wing* (Figs. 4A–C, 5F, 6D)** | | | | | | | | | | | | | | | |
| Wings [shape] | relatively elongated and narrow | elongated, broad | | relatively elongated and narrow | | relatively elongated and narrow | | elongated, broad | | | relatively elongated and narrow | – | relatively elongated | elongated | elongated, broad |
| Distal half of wing [shape] | narrowed, moderately rounded distally | widely rounded distally | | widely rounded distally | | widely rounded distally | | widely rounded distally | | | distinctly narrowed, moderately rounded distally | – | moderately narrowed or widely rounded distally | distinctly narrowed, moderately rounded distally | broadly rounded |
| Costal process [shape] | **prominent, acute apically** | shallow, rounded apically | | moderately prominent, rounded apically | | shallow, rounded apically | | ? shallow, rounded apically | | | **prominent, acute apically** | – | moderately prominent, rounded apically | moderately prominent, rounded apically | shallow, rounded apically |
| Cross veins in the sector of costal process and humeral angle [number] | **1** | up to 7 | | 2–4 | | 4–5 | | ? 2 | | | **1** | – | 1–4 | 2–7 | up to 2 |
| Tornoapical margin [presence of fold] | **absent** | present in MP or between MP_1_ and CuA | | present in MP field or absent | | present [Indistinct] in MP field or absent | | present in MA field | | | **absent** | – | present in MP field or absent | present in MP field or absent | **absent** |
| MA–RSp fork [place of furcation] | **0.12–0.13** | no fork, free RSp basally | | no fork, free RSp basally | | no fork, free RSp basally | | no fork, free RSp basally | | | **0.13** | – | 0.25–0.37 | 0.19–0.21 | no fork, free RSp basally |
| MA fork [presence; shape] | nearly symmetrical | nearly symmetrical | | nearly symmetrical | | nearly symmetrical | | nearly symmetrical | | | symmetrical | – | symmetrical | nearly symmetrical | nearly symmetrical |
| MA fork [place of furcation] | 0.48–0.51 | 0.40–0.43 | | 0.40–0.45 | | 0.48–0.50 | | 0.56 | | | 0.48 | – | 0.50–0.62 | 0.50–0.52 | 0.62–0.64 |
| MP fork [shape] | nearly symmetrical | nearly symmetrical | | nearly symmetrical | | nearly symmetrical | | nearly symmetrical | | | nearly symmetrical | – | nearly symmetrical | nearly symmetrical | nearly symmetrical |
| MP fork [place of furcation] | 0.25–0.27 | 0.22–0.25 | | 0.22–0.24 | | 0.23–0.25 | | 0.22 | | | 0.19 | – | 0.15–0.32 | **0.70–0.75** | **0.75** |
| iMP [shape] | present, long | present, long | | **absent** | | present, long | | present, long | | | present, long | – | present, long | present, long or very short | present, very short |
| Anal venation [longitudinal veins] | rich, well developed | rich, well developed | | rich, well developed | | rich, well developed | | well developed | | | poorly recognizable | – | rich, well developed | rich, well developed | rich, well developed |
| Anal venation  [cross veins] | developed | well developed | | well developed | | well developed | | developed | | | **absent** | – | well developed | rich, well developed | developed |
| Basally connected intercalaries [along tornoapical margin of fore- and hind wings] | developed | rich, well developed | | rich, well developed | | rich, well developed | | developed | | | **poorly developed** | – | rich, well developed | rich, well developed | rich, well developed |
| ***Legs* (Figs. 4D–I)** | | | | | | | | | | | | | | | |
| Hind leg of male [first tarsomere / tibia length ratio] | 0.22–0.25 | 0.16–0.18 | | 0.35–0.40 | | 0.16–0.24 | | – | | | 0.25–0.27 | – | 0.30–0.50 | **0.32–0.38 to 0.72–1.00** | 0.30–0.36 |
| Pretarsal claws | dissimilar | dissimilar | | dissimilar | | dissimilar | | dissimilar | | | dissimilar | – | **similar, pointed** | dissimilar | dissimilar |
| ***Abdominal segments*** | | | | | | | | | | | | | | | |
| Posterolateral projections on segments VII–X | present | present | | present | | absent | | small projections on segment IX only | | | absent | absent | absent | present [moderately to well developed] or absent | absent |
| Subgenital plate of female | present, well developed | present, well developed | | present, well developed | | present, well developed | | present, moderately developed | | | – | present, well developed | present, well developed | **present or absent** | present, well developed |
| Subanal plate of female | present, well developed | present, well developed | | present, well developed | | present, well developed | | moderately developed | | | – | – | present, well developed | **absent** | moderately developed |
| Paracercus [structure and size] | shortened [shorter than cerci] | **subequal to cerci** | | vestigial | | shortened [shorter than cerci] | | strongly shortened to 10 segments | | | – | vestigial  or strongly shortened | shortened or well developed | vestigial or shortened [shorter than cerci] | strongly shortened to 10–12 segments |
| ***Genitalia [male]*** | | | | | | | | | | | | | | | |
| Forceps [number and shape of segments] | three segments; segment I at least 4x longer than segment III | three segments; segment I at least 3x longer than segment III | | **four segments; segment I at least 8x longer than segment IV** | | three segments; segment I at least 4x longer than segment III | | – | | | – | – | three segments; segment I at least 3x longer than segment III | three segments; segment I at least 3–4x longer than segment III | three segments; segment I at least 2.5–3x longer than segment III |

**Remarks**

The Table S1 uses original data as well as information modified from: pp. 410–412, figs. 1, 2 in Tillyard 1921; pp. 271–334, figs. 11–13, 16, 17, 18, 21, 46, 47, 54, 55, 65–68, 76, 77 in Philips 1930; pp. 1–20, figs. 1, 2A, B, 3, 17, 18A–C, 19, 28, 29 in Tillyard 1933; pp. 25 – 29, figs. 1, 2 in Tillyard 1936; pp. 261, figs 69–71 in Harker 1954; pp. 5–9, 11–14, figs 2, 3 in Demoulin 1955; pp. 266–280, figs. 6–9 in Riek 1955; pp. 237–238, fig. 2 in Demoulin 1968; pp. 286–289, figs.16.6A, 16.7B, 16.8B, C, 16.9C in Peters & Campbel 1991; figs. 1, 2 in Domínguez et al. 1994; Kluge et al. 1995; Hitchings & Staniczek 2003; pp. 101–122, figs. 30–36 in Kluge 2004; pp. 151–153, fig. 1 in Mercado & Elliot 2004; pp. 232–234, fig. 2 in Mercado & Elliot 2005; pp. 61–64, fig. 2 in Mercado & Elliot 2006; Domínguez et al. 2006; pp. 2–4, figs. 1–7 in Staniczek & Hitchings 2014; Storari et al. 2023.

For a list of References, see the text of the article.

The female specimen of putative representative of the family Ameletopsidae from the Miocene Dominican amber (Do-1268-K; SMNS collection) is not included since is still undescribed.

Adult characters of *Nebesna* **gen. nov.** are marked in grey. Closest similarities or unique state of important adult characters are marked in bold.

*Abbreviations*: CHA – *Chaquihua* Demoulin, 1955; CHI – *Chiloporter* Lestage, 1931; MIR – *Mirawara* Harker, 1954; AME – *Ameletopsis* Phillips, 1930; BAL – *Balticophlebia* Demoulin, 1968; NEB – *Nebesna* **gen. nov.**; AST – Astraeopteridae Storari et al., 2023; NES – Nesameletidae Riek, 1973; ONI – Oniscigastridae Lameere, 1917; RAL – Rallidentidae Penniket, 1966.

*Other remarks*: †– extinct taxa; * – as preserved; ** – two states of character are referred; PCxsA is complete, i.e. running across ventral side of episternum, clearly separating it on anepisternum [AES] and katepisternum [KES], and terminates reaching sternum; PCxsA is incomplete, i.e. not continued on ventral side of episternum, only partly separating it on anepisternum [AES] and katepisternum [KES], and terminates not reaching sternum (see Kluge et al. 1995; Kluge 2004).

The original Figures cited in the Table S1 are placed in the text of the article.

**References**

Demoulin, G. Ephéméroptères nouveaux ou rares du Chili. II. *Bulletin de l'Institut Royal des Sciences Naturelles de Belgique* **31** (58), 1–16 (1955).

Demoulin, G. Deuxieme contibution à la connaissance des Ephéméroptères de l'ambre oligocene de la Baltique. *Deutsche Entomologische Zeitschrift* **15** (1–3), 233–276 (1968).

Domínguez, E., Hubbard, M. D. & Pescador, M. L. Los Ephemeroptera en Argentina. *Fauna de Agua Dulce de la República Argentina*, *Museo de La Plata* **33** (1), 1–142 (1994).

Domínguez, E., Molineri, C., Pescador, M. L., Hubbard, M. & Nieto, C. *Ephemeroptera of South America*. *Aquatic Biodiversity in Latin America,* ***Volume 2***. (Pensoft, Sofia-Moscow, 1–646, 2006).

Harker, J. E. The Ephemeroptera of Eastern Australia. *Transactions of the Royal Entomological Society of London (B)* **105** (12), 241–268 (1954).

Hitchings, T. R. & Staniczek, A. Nesameletidae (Insecta: Ephemeroptera). *Fauna of New Zealand* **46**, 1–72. <https://doi.org/10.7931/J2/FNZ.46> (2003).

Kluge, N. J. The Phylogenetic System of Ephemeroptera (Kluwer Academic Publishers, Dodrecht, 1–442, 2004).

Kluge, N. J., Studemann, D., Landolt, P., & Gonser, T. A reclassification of Siphlonuroidea (Ephemeroptera). *Mitteilungen der Schweizerischen entomologischen Gesellschaf* **68**, 103–132. <https://doi.org/10.1038/s41598-023-36778-x> (1995).

Mercado, M. & Elliott, S. Taxonomic revision of the genus *Metamonius* Eaton (Nesameletidae: Ephemeroptera), with notes on its biology and distribution. *Studies on Neotropical Fauna and Environment* **39** (2), 149–157. <https://doi.org/10.1080/01650520412331333800> (2004).

Mercado, M. & Elliott, S. Taxonomic revision of the genus *Chiloporter* Lestage (Ameletopsidae: Ephemeroptera) with notes on its biology and distribution. *Studies on Neotropical Fauna and Environment* **40** (3), 229–236. <https://doi.org/10.1080/01650520500140635> (2005).

Mercado, M. & Elliott, S. Taxonomic revision of the genus *Chaquihua* Demoulin (Ameletopsidae: Ephemeroptera) with notes on its biology and distribution. *Studies on Neotropical Fauna and Environment* **41** (1), 59–66. <https://doi.org/10.1080/01650520500475114> (2006).

Peters, W. L. & Campbell, I. C. *Ephemeroptera (Mayflies)*. In Naumann, I. D. & Cane, P. B. (eds). The insects of Australia. (Melbourne Univ. Press & UCL Press Ltd., Melbourne, 279–293, 1991).

Phillips, J. S. Studies of New Zealand mayfly nymphs. *Transactions of the Entomological Society of London* **79**, 399–422 (1930).

Riek, E. F. Revision of the Australian mayflies (Ephemeroptera). I. Subfamily Siphlonurinae. *Australian Journal of Zoology* **3** (2), 266–280 (1955).

Staniczek, A. H. & Hitchings, T. A new species of *Rallidens* (Ephemeroptera: Rallidentidae) from New Zealand. *Records of the Canterbury Museum* **27**, 1–9 (2014).

Storari, A. P., Staniczek, A. H. & Godunko, R. J. A new Gondwanan mayfly family from the Lower Cretaceous Crato Formation, Brazil (Ephemeroptera: Siphlonuroidea: Astraeopteridae fam. nov.). *Scientific Reports* **13**, article number: 11735. https://doi.org/10.1038/s41598-023-36778-x (2023).

Tillyard, R. J. A new genus and species of May-fly (Order Plectoptera) from Tasmania, belonging to the family Siphlonuridae. *Proceedings of the Linnaean Society of New South Wales* **46**, 409–412 (1921).

Tillyard, R. J. The mayflies of Mount Kosciusko region. I. (Plectoptera). Introduction and family Siphlonuridae. *Proceedings of the Linnaean Society of New South Wales* **58**, 1–32 (1933).

Tillyard, R. J. The trout-food insects of Tasmania. Part II. – A monograph of the mayflies of Tasmania. *Papers and Proceedings of the Royal Society of Tasmania* **1935**, 23–59 (1936).
