# Supplementary material for: Nebesna sotnia gen. & sp. nov. from Baltic amber supports a Pangean distribution of the amphinotic family Ameletopsidae (Ephemeroptera)
Source: Sci Rep. 2025 May 26;15:18415. doi: 10.1038/s41598-025-01722-8 (PMC12106746; doi:10.1038/s41598-025-01722-8)
Supplement: Supplementary file 2 — Supplementary Information 2. [file 41598_2025_1722_MOESM2_ESM.docx]

**Supplementary Table S2.** List of comparative adult material of extant species belonging to the families Ameletopsidae Edmunds, 1957, Nesameletidae Riek, 1973, Oniscigastridae Lameere, 1917 and Rallidentidae Penniket, 1966.

| **Taxon / Species name** | **Data on the labels / Database records** | **Ontogenetic**  **stage / sex** | **Inventory number**  **[if present]**  **and collection** |
| --- | --- | --- | --- |
| **Ameletopsidae** | | | |
| *Chaquihua* sp. | Chile: “L.Pe a, 4.1.1968”; “Chile: Prov. Malleco, Rio Bio Bio” | male imago  female imago | ENTU |
| *Ameletopsis perscitus* Eaton, 1899 | New Zealand: “G.F. Edmunds, 6.3.1966”; “Nekon Prov., Gowan Riv., Gowan bridge” | male imago  2 female imagines | ENTU |
| **Nesameletidae** | | | |
| *Nesameletus* sp. | New Zealand: “Penniket, 19.9.1960“; “New Zealand: Huatoki Prov., Kokiri“ | 2 male imagines | ENTU |
| *Nesameletus* sp. | New Zealand: “McLellan, 26.11.1972“; ”New Zealand South Island, Lake Rotoroa Riv. outlet“ | 2 male imagines | ENTU |
| *Nesameletus murihiku* Hitchings & Staniczek, 2003 | New Zealand: 15.12.1992; Halfmoon Bay (Oban); -46,89508/168,114194; 30 m a.s.l.; McIntosh A.R. coll. | male imago | SMNS_EPH_002199_A |
| *-//-* | New Zealand: 18.01.1999; Halfmoon Bay (Oban); -46,89502/168,112884; 50 m a.s.l.; Hitchings T.R. coll. | male imago  female subimago | SMNS_EPH_002200_A  SMNS_EPH_002201_A |
| *Nesameletus austrinus* Hitchings & Staniczek, 2003 | New Zealand: 07.02.1995; Pisgah Spur, Kakanui Mts.; Naseby; -45,06922/170,430162; 1350 m a.s.l.; Patrick B. coll. | male imago | SMNS_EPH_002202_A |
| *-//-* | New Zealand: 12.12.1996; Leith Sole, Waitati; -45,79563/170,512688; 340 m a.s.l.; Patrick B. coll. | male imago | SMNS_EPH_002203_A |
| *-//-* | New Zealand: 19.02.1996; Fox Peak Ski Field, Fairlie; -43,85712/170,815446; 1320 m a.s.l.; Morris S.J. coll. | male subimago | SMNS_EPH_002204_A |
| *Nesameletus vulcanus* Hitchings & Staniczek, 2003 | New Zealand: 25.02.1995; Banks Peninsula, Misty Hills, Little Akaloa; -43,70135/172,962626; 200 m a.s.l.; Ward G.M. coll. | male subimago | SMNS_EPH_002205_A |
| *-//-* | New Zealand: 30.12.1997; Banks Peninsula, Akaroa; -43,82913/173,045843; 25 m a.s.l.; Hitchings T.R. coll. | male imago | SMNS_EPH_002207_A |
| *Nesameletus ornatus* (Eaton, 1883) | New Zealand: 19.10.1993; Mount Thomas Forest, Glentui; -43,20118/172,254201; 320 m a.s.l.; Ward G.M. coll. | male subimago  female subimago | SMNS_EPH_002212_A |
| *-//-* | New Zealand: 13.12.1995; at Mitchell Creek, Maruia; -42,12328/  172,215118; 310 m a.s.l.; Hitchings T.R. coll. | male subimago | SMNS_EPH_002210_A |
| *-//-* | New Zealand: 12.12.1996; Jock Sutton Rd.; Waimahanga; -39,11031/176,685663; 270 m a.s.l.; Crawford W.J. coll. | male subimago | SMNS_EPH_002211_A |
| *-//-* | New Zealand: 13.10.1997; Whiterock; -43,16836/172,510406; 210 m a.s.l.; Hitchings T.R. coll. | male imago  female imago | SMNS_EPH_002208_A  SMNS_EPH_002209_A |
| *Nesameletus flavitinctus* (Tillyard, 1923) | New Zealand: 04.03.1992; Stillwater; -42,42477/171,361863; 30 m a.s.l.; Ward J.B. coll. | female imago | SMNS_EPH_002223_A |
| *-//-* | New Zealand: 20.10.1993; Mount Thomas Forest, Glentui; -43,20118/172,254201; 320 m a.s.l.; Ward J.B. coll. | male imago | SMNS_EPH_002222_A |
| *-//-* | New Zealand: 20.12.1994; Mount Thomas Forest, Glentui; -43,19573/172,246885; 500 m a.s.l.; Hitchings T.R. coll. | male imago | SMNS_EPH_002219_A |
| *-//-* | New Zealand: 30.12.1994; Mount Thomas Forest, Glentui; -43,19573/172,246885; 500 m a.s.l.; Hitchings T.R. coll. | male imago  female imago | SMNS_EPH_002220_A  SMNS_EPH_002221_A |
| *-//-* | New Zealand: 03.10.1995; Smith Ford Br., Nelson; -41,29809/173,342816; 100 m a.s.l.; Ward J.B. coll. | male subimago  female subimago | SMNS_EPH_002224_A |
| *-//-* | New Zealand: 24.04.1997; Tokaanu; Crawford W.J. coll. | female imago | SMNS_EPH_002218_A |
| *-//-* | New Zealand: 28.04.1997; Tokaanu; -38,94415/175,734865; 510 a.s.l.; Crawford W.J. coll. | 2 male imagines | SMNS_EPH_002213_A  SMNS_EPH_002215_A |
| *-//-* | New Zealand: 01.05.1997; Tokaanu; -38,9451/175,732594; 500 m a.s.l.; Crawford W.J. coll. | male subimago | SMNS_EPH_002216_A |
| *-//-* | New Zealand: 18.05.1997; Tokaanu; -38,94415/175,734865; 520 m a.s.l.; Crawford W.J. coll. | female imago | SMNS_EPH_002214_A |
| *-//-* | New Zealand: 22.04.1998; Tokaanu; 520 m a.s.l.; Crawford W.J. coll. | female subimago | SMNS_EPH_002217_A |
| *Nesameletus* sp. | New Zealand: 15.12.1992; Halfmoon Bay (Oban); -46,895078/ 168,114194; 3- m a.s.l.; McIntosh A.R. coll. | female imago | SMNS_EPH_002236_A |
| *-//-* | New Zealand: 30.12.1997; Castle Hill village; -43,14042/171,70174; 1000 m a.s.l.; Hitchings T.R. coll. | female imago | SMNS_EPH_002225_A |
| *-//-* | New Zealand: 30.12.1997; Akaroa; -43,82913/173,045843; 25 m a.s.l.; Hitchings T.R. coll. | female subimago | SMNS_EPH_002231_A |
| *-//-* | New Zealand: 22.01.1997; Fenella Hut - Trilobite Hut, Upper Takaka; -41,06894/172,542072; 920 m a.s.l.; Henderson I.M. coll. | male imago | SMNS_EPH_002234_A |
| *-//-* | New Zealand: 20.02.2002; Canterbury; -42,934337/171,559061; 820 m a.s.l.; Ward G.M. coll. | female imago | SMNS_EPH_005822_A |
| *-//-* | New Zealand: 20.02.1999; Canterbury; -43,14132/171,701721; 1000 m a.s.l.; Hitchings T.R. coll. | female subimago | SMNS_EPH_005825_A |
| **Oniscigastridae** | | | |
| *Oniscigaster distans* Eaton, 1899 | New Zealand: 13.12.1993; Maruia; 410 m. a.s.l.; Hitchings T.R. coll. | male subimago | SMNS_EPH_002406_A |
| *-//-* | New Zealand: 15.02.1995; 4 km below Hut., Otago; -44,895506/168,148744; 594 m a.s.l.; Maturin A.C. coll. | female subimago | SMNS_EPH_005795_A |
| **Rallidentidae** | | | |
| *Rallidens mcfarlanei* Penniket, 1966 | New Zealand: no date and place; Towns D.R. coll. | female imago | SMNS_EPH_000141_A |
| *-//-* | New Zealand; 18.12.1998; Auckland; -36,94024/174,46759; 15 m a.s.l.; Ward G.M. coll. | female imago | SMNS_EPH_005817_A |
| *-//-* | New Zealand; 28.12.1998; Omamari Road, Northland; -35,81295/173,719871; 40 m a.s.l.; Ward G.M. coll. | male imago | SMNS_EPH_005818_A |
| *-//-* | New Zealand; no date; Cascade Stream, Auckland; -36,91512/174,523166; 45 m a.s.l.; Norrie P.H. coll. | male imago | SMNS_EPH_005819_A |
| *-//-* | New Zealand; 08.01.1999; Mangatiore, Northland; -35,17066/173,419081; 110 m a.s.l.; Ward G.M. coll. | female imago | SMNS_EPH_005821_A |
| *-//-* | New Zealand; 14.01.1998; Kouturoa East Rd., Manawatu-Wanganui; -39,10631/175,246172; 200 m a.s.l.; Crawford W.J. coll. | male imago | SMNS_EPH_006236_A |
| *Rallidens platydontis* Staniczek & Hitchings, 2014 | ***Type locality***: 16.02.1998; New Zealand; South Canterbury, Otaio River, tributary, The Hunters Hills; -44,58229/170,885831; Morris S.J. coll. | male imago [***paratype***]  female imago [***paratype***]  2 male imagines  female imago | SMNS_EPH_005810_A  SMNS_EPH_005811_A_001  SMNS_EPH_005811_A_002  SMNS_EPH_005811_A_003  SMNS_EPH_005811_A_004 |
| *-//-* | New Zealand; 07.02.2000; South Canterbury, Otaio River, -44,58775/170,889416; 450 m a.s.l.; collector unknown | female imago | SMNS_EPH_006193_A |

**Acronyms of depositories**

ENTU Institute of Entomology, Biology Centre of the Czech Academy of Sciences, Czech Republic

SMNS Staatliches Museum für Naturkunde Stuttgart, Germany
